# Cost-effectiveness of Intensive vs Standard Blood Pressure Control Among Older Patients With Hypertension

**DOI:** 10.1001/jamanetworkopen.2023.0708

**Published:** 2023-02-27

**Authors:** Chia-Te Liao, Han Siong Toh, Li Sun, Chun-Ting Yang, Angie Hu, Dongmei Wei, Jesus Melgarejo, Zhen-Yu Zhang

**Affiliations:** 1 Studies Coordinating Centre, Research Unit Hypertension and Cardiovascular Epidemiology, KU Leuven Department of Cardiovascular Sciences, University of Leuven, Leuven, Belgium; 2 Department of Public Health, College of Medicine, National Cheng Kung University, Tainan City, Taiwan; 3 Division of Cardiology, Department of Internal Medicine, Chi Mei Medical Center, Tainan City, Taiwan; 4 Department of Intensive Care Medicine, Chi Mei Medical Center, Tainan City, Taiwan; 5 Institute of Clinical Medicine, College of Medicine, National Cheng Kung University, Tainan City, Taiwan; 6 Department of Health and Nutrition, Chia Nan University of Pharmacy and Science, Tainan City, Taiwan; 7 Department of Health Services Research and Policy, London School of Hygiene and Tropical Medicine, London, United Kingdom; 8 Institute of Clinical Pharmacy and Pharmaceutical Sciences, College of Medicine, National Cheng Kung University, Tainan City, Taiwan

## Abstract

**Importance:**

Older patients with hypertension receiving intensive systolic blood pressure control (110-130 mm Hg) have lower incidences of cardiovascular events than those receiving standard control (130-150 mm Hg). Nevertheless, the mortality reduction is insignificant, and intensive blood pressure management results in more medical costs from treatments and subsequent adverse events.

**Objective:**

To examine the incremental lifetime outcomes, costs, and cost-effectiveness of intensive vs standard blood pressure control in older patients with hypertension from the health care payer’s perspective.

**Design, Setting, and Participants:**

This economic analysis was conducted with a Markov model to examine the cost-effectiveness of intensive blood pressure management among patients aged 60 to 80 years with hypertension. Treatment outcome data from the Trial of Intensive Blood-Pressure Control in Older Patients With Hypertension (STEP trial) and different cardiovascular risk assessment models for a hypothetical cohort of STEP-eligible patients were used. Costs and utilities were obtained from published sources. The incremental cost-effectiveness ratio (ICER) against the willingness-to-pay threshold was used to evaluate whether the management was cost-effective. Extensive sensitivity, subgroup, and scenario analyses were performed to address uncertainty. The US and UK population using race-specific cardiovascular risk models were conducted in the generalizability analysis. Data for the STEP trial were collected from February 10 to March 10, 2022, and were analyzed for the present study from March 10 to May 15, 2022.

**Interventions:**

Hypertension treatments with a systolic blood pressure target of 110 to 130 mm Hg or 130 to 150 mm Hg.

**Main Outcomes and Measures:**

Incremental lifetime quality-adjusted life-years (QALYs), costs, and ICER are discounted at the given rates annually.

**Results:**

After simulating 10 000 STEP-eligible patients assumed to be 66 years of age (4650 men [46.5%] and 5350 women [53.5%]) in the model, the ICER values were ¥51 675 ($12 362) per QALY gained in China, $25 417 per QALY gained in the US, and £4679 ($7004) per QALY gained in the UK. Simulations projected that the intensive management in China being cost-effective were 94.3% and 100% below the willingness-to-pay thresholds of 1 time (¥89 300 [$21 364]/QALY) and 3 times (¥267 900 [$64 090]/QALY) the gross domestic product per capita, respectively. The US had 86.9% and 95.6% probabilities of cost-effectiveness at $50 000/QALY and $100 000/QALY, respectively, and the UK had 99.1% and 100% of probabilities of cost-effectiveness at £20 000 ($29 940)/QALY and £30 000 ($44 910)/QALY, respectively.

**Conclusions and Relevance:**

In this economic evaluation, the intensive systolic blood pressure control in older patients produced fewer cardiovascular events and had acceptable costs per QALY gained, well below the typical willingness-to-pay thresholds. The cost-effective advantages of intensive blood pressure management in older patients were consistent over various clinical scenarios across different countries.

## Key Points

**Question:**

Is intensive blood pressure management among older patients with hypertension cost-effective?

**Findings:**

This economic evaluation with a Markov model and extensive sensitivity analyses estimated the incremental lifetime medical costs, quality-adjusted life-years (QALYs), and cost-effectiveness of intensive vs standard blood pressure targets for older patients with hypertension in China, the US, and the UK. Intensive blood pressure control produced fewer cardiovascular events and low costs per QALY gained, well below the typical willingness-to-pay thresholds, and the cost-effective advantages were consistent over various clinical scenarios across different countries.

**Meaning:**

These clinical and economic findings suggest intensive blood pressure control is cost-effective in older adults.

## Teaser

This economic evaluation estimates the lifetime direct medical costs and quality-adjusted life-years of intensive and standard systolic blood pressure treatments among the older population with hypertension in China, the UK, and the US to examine their cost-effectiveness.

## Introduction

Hypertension is a major preventable cause of atherosclerosis, cardiovascular disease (CVD), and mortality.^1,2^ Over the decades, the prevalence has been increasing globally due to the aging population, particularly in low- and middle-income countries.^3^ Consequently, heavy social and financial burdens have been imposed on health care systems.

While the most effective blood pressure treatment target is still unknown, the Systolic Blood Pressure Intervention Trial (SPRINT trial) demonstrated that intensive systolic blood pressure (SBP) control (target, <120 mm Hg) contributes to more clinical cardiovascular benefits, lower mortality, and better cost-effectiveness than standard control (target, <140 mm Hg).^4,5,6^ However, recommendations for blood pressure goals still vary widely according to different guidelines and age groups.^7,8,9^

The 2019 guidelines for hypertension management among older individuals in China defined the hypertension thresholds as SBP of at least 140 mm Hg and/or diastolic blood pressure of at least 90 mm Hg.^10^ Recently, the Trial of Intensive Blood-Pressure Control in Older Patients With Hypertension (STEP trial)^11^ showed significant incidence reductions of cardiovascular events with intensive SBP treatments (target, 110-130 mm Hg) vs standard treatments (target, 130-150 mm Hg) among patients aged 60 to 80 years with hypertension and at high CVD risk in China. Although the intensive treatments reduce cardiovascular events compared with the standard treatments, they may simultaneously lead to more adverse events resulting from lower blood pressure and higher medical costs due to increased use of antihypertensive agents, clinic visits, and laboratory monitoring. Moreover, a shorter duration of antihypertensive use, higher mortality as a competing risk for CVD, and adherence may influence the health-economic incentive.^12,13^ Therefore, this study aimed to estimate the lifetime direct medical costs and quality-adjusted life-years (QALYs) of intensive and standard SBP treatments among the older population with hypertension in different countries to examine the cost-effectiveness.

## Methods

This economic evaluation was deemed exempt from review and informed consent by the Institutional Review Board of Chi Mei Medical Center because it was not considered human participants research. This study was reported following the Consolidated Health Economic Evaluation Reporting Standards (CHEERS) reporting guideline.

### Statistical Analysis

#### Model Structure and Assumption

**Figure 1.**
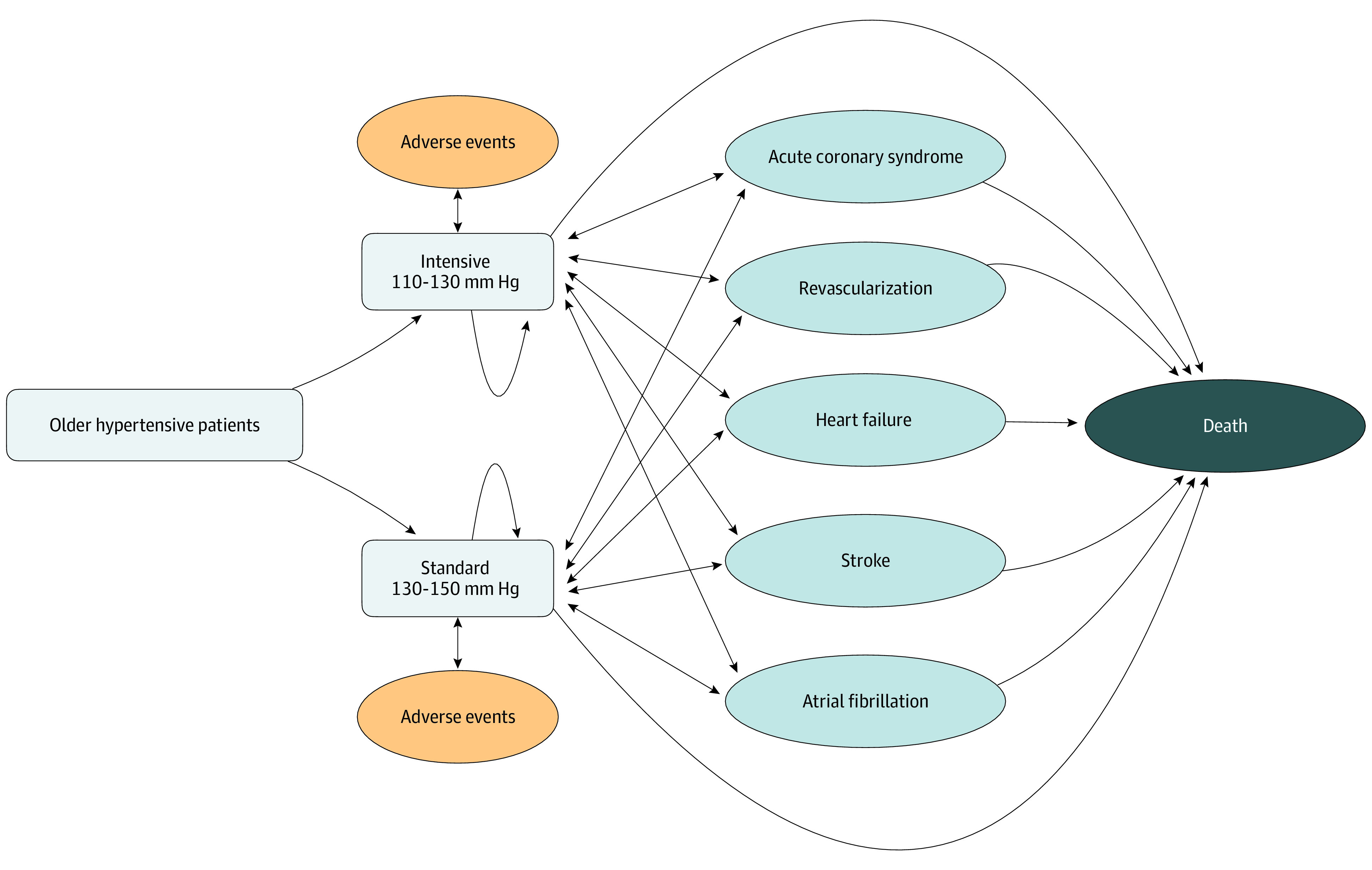
Conceptual Diagram of the Cost-effectiveness Model Based on the Trial of Intensive Blood Pressure Control in Older Patients With Hypertension (STEP) Older patients with hypertension were assigned to 2 different strategies of blood pressure control. The structure of the hypertension model included 6 health states (no cardiovascular disease events, chronic coronary heart disease, post stroke, chronic heart failure, chronic atrial fibrillation, and death) and 2 main clinical events (adverse events and cardiovascular events). Patients in each treatment strategy remained stable with noncardiovascular events, developed cardiovascular diseases, or died according to the transitional probabilities. Adverse events, medical costs, and quality-adjusted life-year accrued on the hypertension treatments.

Data for the STEP trial used for simulation were collected from February 10 to March 10, 2022, and analyzed for the present study from March 10 to May 15, 2022. We constructed a Markov model to simulate 10 000 STEP-eligible patients with hypertension receiving intensive and standard blood pressure control and estimated the lifetime direct medical costs, cardiovascular events, QALYs, and incremental cost-effectiveness ratio (ICER).^11^ According to the trial, the starting age for the simulated patients was assumed to be 66 years, and the model used 1 year as each cycle length. All simulated patients progressed from hypertension without acute events through the Markov model until death. The structure of the hypertension model from previous studies was adopted^6,13,14^ and included 6 health states and 2 main clinical events (Figure 1 and eFigures 1 and 2 in Supplement 1). Cardiovascular events included acute coronary syndrome, acute stroke, coronary revascularization, acute heart failure, atrial fibrillation attack, and cardiovascular death. The adverse events of interest included hypotension, dizziness, syncope, fracture, and acute kidney injury.

This model compared the lifetime cost-effectiveness of intensive vs standard blood pressure treatments from the health care payer’s perspective in different countries. Apart from Han people in China, the analyses were conducted in the US and the UK to consider the generalizability of different health care systems. Future medical costs and utility were discounted at 3.0% in China and the US and 3.5% in the UK according to the previous studies or the given guideline recommendations.^6,15,16^ All analyses were conducted on Excel, version 16 (Microsoft Corporation), and TreeAge Pro, version 2021 (TreeAge Software).

#### Probability of Clinical Events

We extracted the yearly transitional probabilities from the STEP trial for cardiovascular events, treatment-related adverse events, cardiovascular death, and all-cause mortality during the 4-year trial period (Table 1). The repeated CVD risks were assumed to be consistent with the estimates of first-time CVD events, which varied in scenario analyses to address the uncertainty. As the simulated patients were older, the equations of risk assessment models (ie, Systematic Coronary Risk Evaluation 2 [SCORE2] and SCORE2–Older Patients [SCORE2-OP]) were used to provide more appropriate estimations for the older population after the 4-year trial period^17,18^ (eMethods in Supplement 1). Different CVD risk assessment models were also applied in scenario analyses. The yearly mortality risks were extracted from the individual national age-specific mortality statistical database to account for the competing risk of death from non-CVD causes.^19,20,21^

**Table 1.** Selected Input Estimates, SDs, and Distribution for the Transitional Probability of Clinical Events in the Simulation Model

Cardiovascular event	Estimates (yearly probability)^a^	SD	Distribution
**Primary outcome^b^**			
Intensive control	0.008 776 182	0.001 431 865	Beta
Standard control	0.011 683 970	0.001 644 868	Beta
Relative risk	0.751 130 175	0.215 494 892	Log-normal
Stroke			
Intensive control	0.002 840 265	0.000 817 006	Beta
Standard control	0.004 185 055	0.000 988 161	Beta
Acute coronary syndrome			
Intensive control	0.003 256 504	0.000 874 643	Beta
Standard control	0.004 838 185	0.001 062 126	Beta
Acute heart failure			
Intensive control	0.000 299 955	0.000 265 844	Beta
Standard control	0.000 899 595	0.000 458 898	Beta
Coronary revascularization			
Intensive control	0.000 999 500	0.000 485 107	Beta
Standard control	0.001 998 001	0.000 683 520	Beta
Atrial fibrillation			
Intensive control	0.001 417 103	0.000 577 506	Beta
Standard control	0.001 467 614	0.000 585 969	Beta
Cardiovascular death			
Intensive control	0.001 062 262	0.000 500 090	Beta
Standard control	0.001 467 614	0.000 585 969	Beta
**Adverse events**			
Hypotension			
Intensive control	0.008 715 688	0.001 426 965	Beta
Standard control	0.006 685 776	0.001 247 404	Beta
Dizziness			
Intensive control	0.002 662 037	0.000 791 028	Beta
Standard control	0.002 882 637	0.000 820 646	Beta
Syncope			
Intensive control	0.000 353 711	0.000 288 676	Beta
Standard control	0.000 117 171	0.000 165 681	Beta
Fracture			
Intensive control	0.000 884 983	0.000 456 498	Beta
Standard control	0.001 114 796	0.000 510 791	Beta
Acute kidney injury			
Intensive control	0.003 386 435	0.000 909 393	Beta
Standard control	0.003 724 914	0.000 949 418	Beta
All-cause death^c^			
Intensive control	0.003 971 272	0.000 965 527	Beta
Standard control	0.003 770 095	0.000 938 088	Beta

^a^ The yearly transition probabilities were transformed as follows: (1) probability (obtained from the Trial of Intensive Blood-Pressure Control in Older Patients With Hypertension) transformed to the rate of [−ln (1 − p)] / t and (2) rate transformed to a probability (yearly transition probability applied in the analyses): 1 − exp(−rt), where r is the rate, p is the probability, and t is the time.

^b^ After the trial period, the probability of cardiovascular events was derived from the cardiovascular risk assessment models.

^c^ After the trial period, the input parameter was extracted from the life table in different countries.

Adherence is crucial in reaching the target for blood pressure management. After the trial period, the treatment outcomes changed with adherence, age, and SBP values. Three scenarios were then used to simulate the dynamic outcomes of the 2 treatment strategies on a lifetime horizon. For the base-case analyses, adherence to intensive and standard treatments was based on the percentages of the reached target at the end of the STEP trial and the number of antihypertensive medications (ie, more medications are associated with lower adherence).^11,22,23^ In the worst-case scenario, patients in the intensive group were assumed to be nonadherent to antihypertensive medications immediately after the trial. The standard group had complete adherence and obtained the corresponding treatment outcomes. In the best-case scenario, patients in the intensive group adhered to all medications; the standard group adhered to medications as in the base case (eMethods in Supplement 1).

#### Cost and Utility Estimates

We estimated the lifetime direct medical costs of the simulated patients, including annual costs of blood pressure control, acute cardiovascular and adverse events, chronic cardiovascular events, and background health care costs for non-CVD events (eTables 3, 7, and 11 in Supplement 1). Annual medical intervention costs comprised antihypertensive medications, clinic visits, and laboratory monitoring. Costs of antihypertensive medications were calculated by weighting the mean of the generic formulary medications in the STEP trial and the published drug costs in each country (eMethods and eTables 3, 7, and 11 in Supplement 1). The frequency and the fees for physicians, nursing staff, and laboratory operations were taken into account to estimate the clinic visits and monitoring. Other medical costs of acute events and chronic states were derived from the national database or published studies (eTables 3, 7, and 11 in Supplement 1). All cost inputs were inflated to 2022 and converted to the given country’s currency (Chinese yuan renminbi [¥], US dollar [$], and UK pound sterling [£]) by the currency rates of purchasing power parities^24^ (eMethods in Supplement 1).

Utility values range from 0 (death) to 1 (perfectly healthy without disability) for different health states and clinical events. We extracted the values from the results of the EuroQol Group 5-Dimension Self-Report Questionnaire in the published literature, considering the appropriateness of the study population (eg, race and ethnicity, population with disease) and the quality and comprehensiveness of the source. Utility decrements were used for age, coronary heart disease, acute kidney injury for 4 weeks, fracture for 12 weeks, and other adverse events for 2 weeks. Detailed cost and utility inputs are shown in eTables 3, 7, and 11 in Supplement 1.

#### Model Validation

We used 2 methods to validate the model. First, we compared the predicted and observed primary outcomes and other cardiovascular events and percentages during the STEP trial. Second, we compared the cumulative incidence of primary outcomes with a visual inspection (ie, cumulative incidence curves between model predictions and STEP observations).

#### Base-Case Cost-effectiveness Analysis

The model was run with the time horizon of a lifetime. Lifetime medical costs were calculated by multiplying the number of patients with the sum of the costs in each health status. Total QALYs were accumulated from the QALY in each cycle, obtained from the utility values associated with each health status multiplied by the proportion of patients living in that status. The ICER was calculated by dividing the incremental costs by the incremental QALYs (ie, costs per QALY gained). We applied the willingness-to-pay thresholds of ¥89 300 ($21 364)/QALY and ¥267 900 ($64 090)/QALY in China, $50 000/QALY and $100 000/QALY in the US, and £20 000 ($29 940)/QALY and £30 000 ($44 910)/QALY in the UK to determine the cost-effectiveness of, respectively, standard vs intensive treatments in older patients with hypertension.

#### Sensitivity Analysis

We performed 1-way sensitivity analyses and probabilistic sensitivity analyses (PSA) to quantify the variations in ICER values caused by parameter uncertainties. One-way sensitivity analyses with varying values for all input parameters through plausible ranges (±10%) were used to examine the individual impact on the ICER values. The results are presented as a tornado diagram in eFigures 5, 8, and 11 in Supplement 1. The Monte Carlo simulation was run 1000 times in the PSA with random draws from predefined uncertainty distributions of all model inputs.

#### Scenario and Subgroup Analyses

Scenario analyses examined the impact of various assumptions and inputs with great influence in 1-way sensitivity analyses, including medication adherence (gradually decreasing and persistent); costs including intervention, acute events, and chronic states; treatment outcomes; different discount rates (0% and 5%); the risk of adverse events and cardiovascular events; repeated CVD risk; and different time horizons. Moreover, in the trial period, different treatment outcomes for the primary outcome were input to account for generalization in non-Chinese settings (eg, the hazard ratio of 0.68 for the older patients in the SPRINT trial). After the trial period, the scenario analyses additionally used different CVD risk assessment models to simulate the CVD probabilities to examine the robustness of external generalizability (ie, the Prediction for ASCVD [Atherosclerotic CVD] Risk in China [China-PAR]^25^ and the American Heart Association–American College of Cardiology [AHA-ACC] pooled cohort equation^26^ models) (eTables 4 and 8 in Supplement 1). All-cause mortality was a competing risk in the model; the mortality was thus assessed in the scenario analyses.

We analyzed the cost-effectiveness in subgroups stratified by age (60-69 and 70-80 years), sex (men and women), distribution of SBP upon enrollment (≤138 mm Hg, 139-151 mm Hg, and ≥152 mm Hg), previous diabetes (presence and absence), and types of blood pressure measurement (using a phone app and usual managements) according to the subgroup analyses in the STEP trial.

## Results

### Model Validation

A total of 10 000 hypothetical STEP-eligible patients (assumed to be 66 years of age; 4650 men and 5350 women) were simulated in this model. After 4 years of simulation, the incidence rates of the primary outcome between the model and the STEP trial were estimated to be 1.14 vs 1.00 per 100 person-years for intensive treatment and 1.39 vs 1.40 per 100 person-years for standard treatment. The model projected similar cumulative incidence curves of primary outcomes for the intensive and standard treatments as the STEP trial (eFigure 3 and eTable 1 in Supplement 1).

### Base-Case, Best-case, and Worst-case Analyses

After lifetime simulation, the rates of cardiovascular events in the UK and the US were projected to be higher than in China, while the rate of noncardiovascular death in China was the highest. The base-case analysis projected the intensive treatments to avert 353 cardiovascular events per 1000 patients over the lifetime in China, 325 per 1000 patients in the US, and 428 per 1000 patients in the UK, compared with standard treatments. The worst-case analysis was estimated to prevent 136 and 154 cardiovascular events per 1000 patients in China and the UK, respectively, but the benefit was not obvious in the US (eTable 2 in Supplement 1).

In base-case analyses, intensive blood pressure treatments in the US were projected to produce the greatest incremental QALYs (0.29) and related medical costs ($7371). The ICER value in the UK was estimated to be the lowest (£4679 [$7004] per QALY gained), followed by China (¥51 675 [$12 362] per QALY gained) and the US ($25 417 per QALY gained). The best-case and worst-case analyses projected the ICERs to be £3434 ($5141) and £8588 ($12 856) per QALY gained in the UK, respectively; ¥43 951 ($10 515) and ¥71 232 ($17 041) per QALY gained in China, respectively; and $20 748 and $40 608 per QALY gained in the US, respectively (Table 2).

**Table 2.** Cost-effectiveness of Intensive vs Standard Blood Pressure Treatment in Older Populations With Hypertension Across Different Countries^a^

Scenario	QALYs			Costs			ICER, costs per QALY gained	Probability of cost-effectiveness at different WTP thresholds, %	
	Intensive	Standard	Difference (95% UI)	Intensive	Standard	Difference (95% UI)		Lower	Upper
**China**									
Base case	9.53	9.29	0.24 (0.23-0.24)	¥111 046 ($26 566)	¥98 644 ($23 599)	¥12 402 ($2967) (¥10 007-¥15 439 [$2394-$3694])	¥51 675 ($12 362)	94.3	100
Worst case	9.50	9.30	0.20 (0.19-0.21)	¥112 957 ($27 023)	¥98 711 ($23 615)	¥14 246 ($3408) (¥11 273-¥16 641 [$2696-$3981])	¥71 232 ($17 041)	74.1	99.7
Best case	9.55	9.28	0.27 (0.26-0.28)	¥110 406 ($26 413)	¥98 539 ($23 574)	¥11 867 ($2839) (¥9451-¥14 860 [$2261-$3555])	¥43 951 ($10 515)	96.4	100
**US**									
Base case	10.95	10.66	0.29 (0.28-0.30)	$219 671	$212 300	$7371 ($5673-$9320)	$25 417	86.9	95.6
Worst case	10.91	10.67	0.24 (0.23-0.26)	$221 716	$211 970	$9746 ($7767-$11 606)	$40 608	63.2	85.9
Best case	10.97	10.66	0.31 (0.29-0.35)	$218 732	$212 300	$6432 ($4897-$8626)	$20 748	90.7	97.1
**UK**									
Base case	10.11	9.83	0.28 (0.27-0.29)	£72 081 ($107 906)	£70 771 ($105 945)	£1310 ($1961) (£893-£1578 [$1337-$2362])	£4679 ($7004)	99.1	100
Worst case	10.07	9.83	0.24 (0.23-0.24)	£72 817 ($109 007)	£70 756 ($105 922)	£2061 ($3085) (£1550-£2567 [$2320-$3843])	£8588 ($12 856)	83.7	100
Best case	10.12	9.83	0.29 (0.28-0.30)	£71 767 ($107 436)	£70 771 ($105 945)	£996 ($1491) (£555-£1769 [$831-$2648])	£3434 ($5141)	99.5	100

Abbreviations: ICER, incremental cost-effectiveness ratio; QALY, quality-adjusted life-year; UI, uncertainty interval; WTP, willingness-to-pay.

^a^ The adherence rates of intensive vs standard treatments were assumed at 70% and 75% for base-case, 0% and 100% for worst-case, and 100% and 75% for best-case scenarios. Willingness-to-pay thresholds of 1 and 3 times the gross domestic product per capita based on purchasing power parities (lower, ¥89 300 [$21 364]; upper, ¥267 900 [$64 090]) were used in China. The thresholds in the US (lower, $50 000; upper, $100 000) and UK (lower, £20 000 [$29 940]; upper, £30 000 [$44 910]) were based on individual guideline recommendations.

### One-way and Probabilistic Sensitivity Analyses

One-way sensitivity analyses showed the association of uncertainty ranges of individual variables with cost-effectiveness (eFigures 5, 8, and 11 in Supplement 1). In China, the ICER changes were estimated at ±¥14 931 ($3572) (±21.8%). The model was most sensitive to yearly costs of blood pressure treatments, risk of primary outcomes, risk and utility of stroke, and risk of acute coronary syndrome. In the US, the ICER changes were projected from −$9696 (−38.1%) to +$15 617 (+61.4%). The model was most sensitive to the risk of primary outcomes, probability of noncardiovascular death, yearly costs of blood pressure treatments, risk of stroke and acute coronary syndrome, and utility of stroke. In the UK, the variables associated with the greatest ICER changes included probability of noncardiovascular death, risk of primary outcomes, yearly costs of blood pressure treatments, and risk of stroke and acute coronary syndrome (ie, from −£12 774 [$19 123 (−273.1%)] to +£4479 ([$6705 (+95.7%)]).

**Figure 2.**
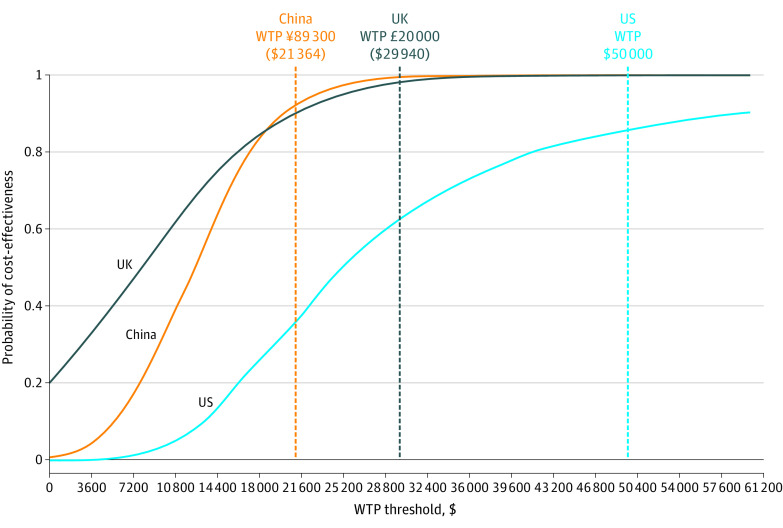
Probability of Cost-effectiveness of Intensive vs Standard Blood Pressure Control in China, the US, and the UK The curve presents the results after running 1000 simulations with random draws for all input parameters to capture joint uncertainty and the probabilities of cost-effectiveness of intensive treatments, which changed with different willingness-to-pay (WTP) thresholds (costs in US dollars per quality-adjusted life-year gained).

**Figure 3.**
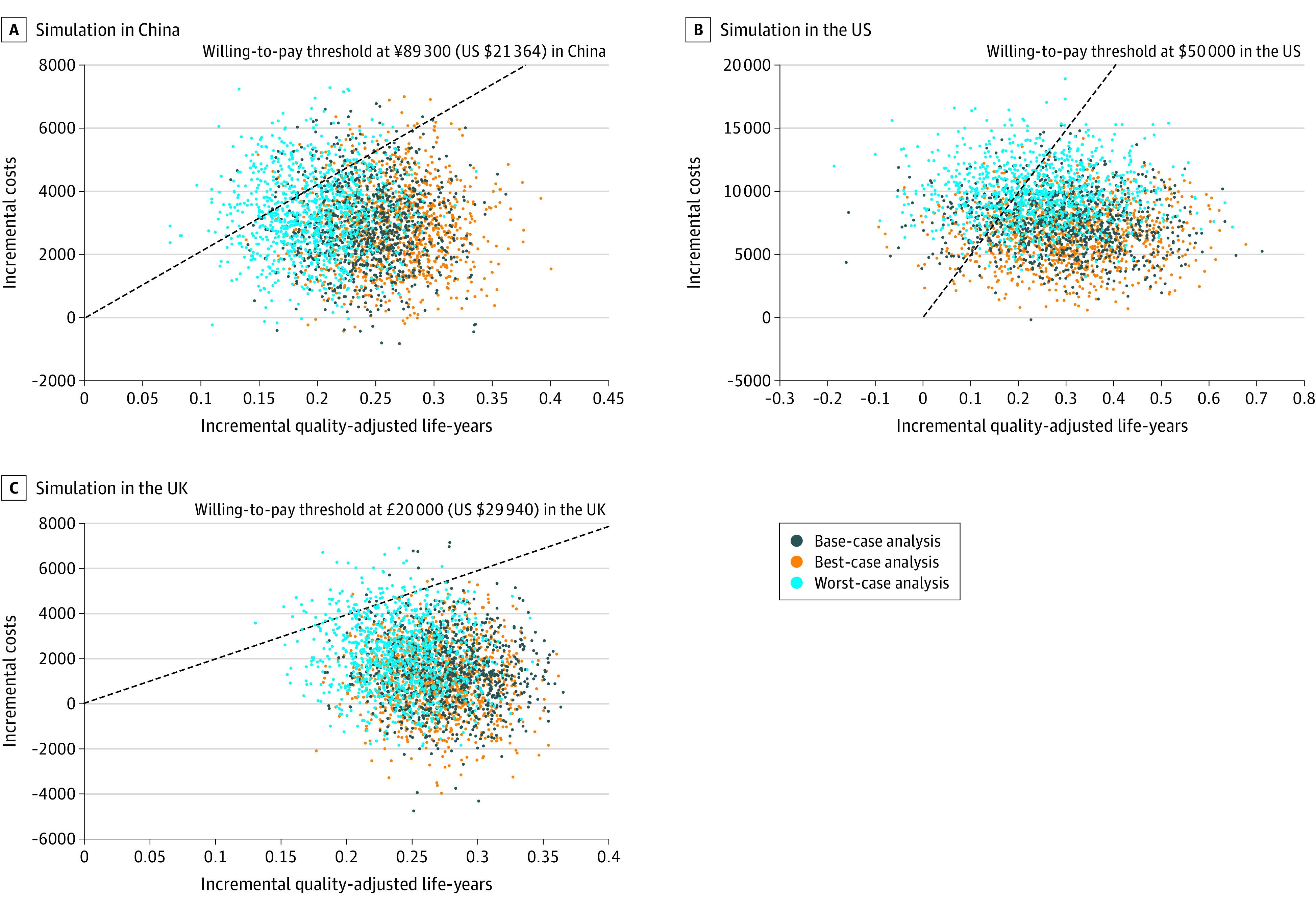
Cost-effectiveness of Incremental Costs and QALYs The cost-effectiveness planes show the results of probabilistic sensitivity analyses in scenarios with different adherence (base case, worst case, and best case). Every plot presents the incremental costs and quality-adjusted life-years between intensive and standard blood pressure control among older patients with hypertension in each simulation. The northeast quadrant represents more effective and more costly treatment. Most incremental cost-effectiveness ratio plots are located in this area, and those under the willingness-to-pay line represent cost-effectiveness. The plots in the southeast quadrant indicate cost savings due to being more effective but less costly.

In base-case PSA, the UK was estimated to have the highest probability (99.1%) of intensive treatments being cost-effective at a willingness-to-pay threshold of £20 000 ($29 940)/QALY; China was estimated to be 94.3% at ¥89 300 ($21 364)/QALY and the US to be 86.9% at $50 000/QALY (Figure 2). The best-case PSA projected all the probabilities to be higher than 90%, while the probabilities in the UK decreased to 83.7%, in China to 74.1%, and in the US to 63.2% in the worst-case PSA (Table 2). At the extended willingness-to-pay thresholds, the probabilities were projected to be higher than 90% regardless of base-case, best-case, or worst-case scenarios (eFigures 4, 7, and 10 in Supplement 1). Cost-effectiveness planes visually represent the incremental costs and QALYs between the 2 treatments in Figure 3. Notably, the UK was estimated to have the highest cost-saving probability (ie, most spots in the southeast quadrant).

### Scenario and Subgroup Analyses

Thirty-six scenario analyses were performed in the individual countries. The costs of blood pressure management and time horizon had greater effects on the base-case ICERs (eTables 5, 9, and 12 in Supplement 1). Adherence, treatment outcomes, CVD risk prediction models, risks of CVD and adverse events, all-cause mortality rates, and discount rates had smaller effects on the ICER values.

Subgroup analyses showed similar cost-effectiveness in individual stratifications. Men treated with an intensive target had lower ICERs than women. Intensive treatments in patients aged 60 to 69 years had lower ICERs in China and the UK, while intensive treatments in those aged 70 to 80 years had lower ICER values in the US (eTables 6, 10, and 13 and eFigures 6, 9, and 12 in Supplement 1).

## Discussion

This economic evaluation simulated STEP-eligible patients to estimate the cost-effectiveness of intensive and standard management. Although greater medical costs for blood pressure control, adverse events, shorter life expectancy, and treatment durations may reduce the economic incentive, the prevention of cardiovascular events provided significant benefits in cost-effectiveness. The ICERs in China, the UK, and the US were all lower than the willingness-to-pay thresholds, and the probabilities of intensive treatments being cost-effective were higher than 60% even in the worst-case analyses. The positive results of scenario analyses strengthened the cost-effectiveness of intensive treatments for older patients with hypertension from the health care payer’s perspective in different settings (eg, a developing country with multilevel medical security systems, a developed country with the governmental universal health care system or mixed public-private health coverage, and even a country with remarkably higher medical costs).

Due to different races, the STEP-eligible simulation may lead to uncertainty in the US and UK. During the trial period, we thus applied different treatment outcomes for older patients. After the trial, we used various CVD risk models to consider the uncertainty. Higher estimated CVD risks resulted in better cost-effectiveness (ie, AHA-ACC pooled cohort equation >SCORE2 and SCORE2-OP >China-PAR models). Moreover, the countries’ age-specific mortality rates were applied to address the uncertainty. We found that the country with lower all-cause mortality and longer life expectancy has better cost-effectiveness.

The SPRINT and SPRINT-based cost-effectiveness analyses have provided clinical benefits and good cost-effectiveness.^4,5,6,12,14^ Nevertheless, the concerns of an intensive target in older patients (SBP <120 mm Hg) may lead to inconsistent SBP targets (ie, 150 mm Hg for the American College of Physicians–American Academy of Family Physicians, 140 mm Hg for the Chinese Geriatrics Society, 130 to 139 mm Hg for the European guidelines, <130 mm Hg for the AHA-ACC guidelines).^7,8,9,10^ Compared with SPRINT, despite lacking mortality reduction, less intensive blood pressure control (mean, 130 mm Hg in the STEP trial) for older patients still reduces cardiovascular incidence and showed cost-effective advantages. The study by Xie et al^27^ set in China projected more favorable ICERs at ¥7876 ($1884) per QALY gained, which was much lower than the estimation in another study (¥38 929 [$9313])^6^ and our study (¥51 675 [$12 362]). This may be due to inflation, different blood pressure targets and relevant costs, the 10-year model duration, a younger generation, the lack of background treatment costs, and neglect of atrial fibrillation and heart failure events in the model. In the US, Richman et al^14^ and Bress et al^13^ projected that the ICERs for intensive hypertensive control (<120 mm Hg) were $23 777 and $46 546 per QALY gained, respectively. Notably, the subgroup analyses of Bress et al^13^ yielded more favorable ICERs ($26 000 per QALY gained) for older patients, similar to our estimates. However, the less intensive treatments may alleviate the concerns and provide more incentive to implement consistent blood pressure targets for older patients.

### Limitations

Our study has some limitations. First, our analyses extracted parameters from the STEP trial, but the trial only enrolled Han people, which may cause uncertain generalizability. Thus, the scenario analyses used various treatment outcomes during the trial period and different race- and country-specific CVD risk models after the trial period. Although the models and treatment outcomes could not fit all scenarios perfectly, the various analyses provided ranges of uncertainty. These consistently positive results strengthened the generalizability and robustness of cost-effectiveness analyses. Second, the study did not present repeated CVD risks. We simplified the realistic situation without excessive extrapolation to closely model the simulation’s STEP trial findings. The assumptions and estimations were conservative. In our scenario analyses, repeated CVD risks that were doubled or higher led to more favorable ICERs. In this case, intensive hypertension management for older patients would be more cost-effective than our estimations. Third, this model did not include all hypertension-related events. Despite various scenario analyses, it is impossible to cover all hypertension-mediated results. For example, we did not consider patients who progressed to end-stage kidney diseases from acute kidney injury. In the STEP trial, the risks of kidney deterioration between the intensive and standard groups did not differ significantly, even for those without chronic kidney diseases at baseline. Besides, the long-term kidney outcomes of the STEP participants were still lacking. Therefore, our model did not include kidney outcomes to avoid excessive uncertainty. Given the kidney outcomes of post hoc analyses from the STEP trial, future cost-effectiveness studies may need to consider long-term kidney-relevant costs and quality of life.

## Conclusions

In this economic evaluation of intensive (110-130 mm Hg) vs standard (130-150 mm Hg) SBP control in older hypertensive patients with high CVD risks, the intensive treatments produced fewer cardiovascular events and low costs per QALY gained, commonly below the willingness-to-pay thresholds. The cost-effectiveness was consistently favorable across various clinical scenarios in different countries. These clinically and economically promising findings may bridge the gap between the trial and guideline recommendations in future hypertension prevention and treatments.

## Notes

Supplement 1. **eMethods.** Hypothetical Cohort and Models **eFigure 1.** Detailed Markov Model Including 2 Treatment Strategies, 6 Health States, Adverse Events, and Cardiovascular Events **eFigure 2.** Example of the Markov Model Simulation in This Study **eFigure 3.** Cumulative Incidence of Primary Cardiovascular Outcomes Between Intensive and Standard Blood Pressure Control in Older Patients in the Simulated Model and the STEP Trial **eTable 1.** Comparison of the Percentage of Cardiovascular Events in the Model After the Trial-Period Simulation and in the STEP Trial **eTable 2.** Cardiovascular Events per 1000 Patients With Intensive and Standard Treatments After Lifetime Simulation in Different Settings **eTable 3.** Utility and Cost Parameters in the Model in the Chinese Setting **eTable 4.** Yearly Cardiovascular Disease Risk Extracted From Different 10-Year Cardiovascular Risk Prediction Models in the Chinese Setting **eFigure 4.** Probability of Cost-effectiveness of Intensive vs Standard Blood Pressure Control in Different Adherence Scenarios in China **eFigure 5.** One-way Sensitivity Analysis Presented as a Tornado Diagram for the Chinese Setting **eTable 5.** Assumptions in the Model and Relevant Scenario Analyses in the Chinese Setting **eTable 6.** Cost-effectiveness of Intensive vs Standard Blood Pressure Control Among Older Patients With Different Stratifications in China **eFigure 6.** Probability of Cost-effectiveness of Intensive vs Standard Blood Pressure Control by Age, Sex, and Systolic Blood Pressure at Baseline in China **eTable 7.** Utility and Cost Parameters Simulated in the Model for the US Setting **eTable 8.** Yearly Cardiovascular Disease Risk Extracted From Different 10-Year Cardiovascular Risk Prediction Models in the US Setting **eFigure 7.** Probability of Cost-effectiveness of Intensive vs Standard Blood Pressure Control in Different Adherence Scenarios in the US **eFigure 8.** One-way Sensitivity Analysis Presented as a Tornado Diagram for the US Setting **eTable 9.** Assumptions in the Model and Relevant Scenario Analyses in the US Setting **eTable 10.** Cost-effectiveness of Intensive vs Standard Blood Pressure Control Among Older Patients With Different Stratifications in the US **eFigure 9.** Probability of Cost-effectiveness of Intensive vs Standard Blood Pressure Control by Age, Sex, and Systolic Blood Pressure at Baseline in the US **eTable 11.** Utility and Cost Parameters Simulated in the Model for the UK Setting **eFigure 10.** Probability of Cost-effectiveness of Intensive vs Standard Blood Pressure Control in Different Adherence Scenarios in the UK **eFigure 11.** One-way Sensitivity Analysis Presented as a Tornado Diagram for the UK Setting **eTable 12.** Assumptions in the Model and Relevant Scenario Analyses in the UK Setting **eTable 13.** Cost-effectiveness of Intensive vs Standard Blood Pressure Control Among Older Patients With Different Stratifications in the UK Setting **eFigure 12.** Probability of Cost-effectiveness of Intensive vs Standard Blood Pressure Control by Age, Sex, and Systolic Blood Pressure at Baseline in the UK
eReferences

Supplement 2.
Data Sharing Statement
