# Supplementary material for: Cost-effectiveness of Intensive vs Standard Blood Pressure Control Among Older Patients With Hypertension
Source: JAMA Netw Open. 2023 Feb 27;6(2):e230708. doi: 10.1001/jamanetworkopen.2023.0708 (PMC9972197; doi:10.1001/jamanetworkopen.2023.0708)
Supplement: Supplement 1. — eMethods. Hypothetical Cohort and Models eFigure 1. Detailed Markov Model Including 2 Treatment Strategies, 6 Health States, Adverse Events, and Cardiovascular Events eFigure 2. Example of the Markov Model Simulation in This Study eFigure 3. Cumulative Incidence of Primary Cardiovascular Outcomes Between Intensive and Standard Blood Pressure Control in Older Patients in the Simulated Model and the STEP Trial eTable 1. Comparison of the Percentage of Cardiovascular Events in the Model After the Trial-Period Simulation and in the STEP Trial eTable 2. Cardiovascular Events per 1000 Patients With Intensive and Standard Treatments After Lifetime Simulation in Different Settings eTable 3. Utility and Cost Parameters in the Model in the Chinese Setting eTable 4. Yearly Cardiovascular Disease Risk Extracted From Different 10-Year Cardiovascular Risk Prediction Models in the Chinese Setting eFigure 4. Probability of Cost-effectiveness of Intensive vs Standard Blood Pressure Control in Different Adherence Scenarios in China eFigure 5. One-way Sensitivity Analysis Presented as a Tornado Diagram for the Chinese Setting eTable 5. Assumptions in the Model and Relevant Scenario Analyses in the Chinese Setting eTable 6. Cost-effectiveness of Intensive vs Standard Blood Pressure Control Among Older Patients With Different Stratifications in China eFigure 6. Probability of Cost-effectiveness of Intensive vs Standard Blood Pressure Control by Age, Sex, and Systolic Blood Pressure at Baseline in China eTable 7. Utility and Cost Parameters Simulated in the Model for the US Setting eTable 8. Yearly Cardiovascular Disease Risk Extracted From Different 10-Year Cardiovascular Risk Prediction Models in the US Setting eFigure 7. Probability of Cost-effectiveness of Intensive vs Standard Blood Pressure Control in Different Adherence Scenarios in the US eFigure 8. One-way Sensitivity Analysis Presented as a Tornado Diagram for the US Setting eTable 9. Assumptions in the Model and Relevant Scena [file jamanetwopen-e230708-s001.pdf]

Supplementary Online Content

Liao CT, Toh HS, Sun L, et al. Cost-effectiveness of intensive vs standard blood pressure control among older patients with hypertension. *JAMA Netw Open*. 2023;6(2):e230708. doi:10.1001/jamanetworkopen.2023.0708

eMethods. Hypothetical Cohort and Models
eFigure 1. Detailed Markov Model Including 2 Treatment Strategies, 6 Health States, Adverse Events, and Cardiovascular Events
eFigure 2. Example of the Markov Model Simulation in This Study
eFigure 3. Cumulative Incidence of Primary Cardiovascular Outcomes Between Intensive and Standard Blood Pressure Control in Older Patients in the Simulated Model and the STEP Trial
eTable 1. Comparison of the Percentage of Cardiovascular Events in the Model After the Trial-Period Simulation and in the STEP Trial
eTable 2. Cardiovascular Events per 1000 Patients With Intensive and Standard Treatments After Lifetime Simulation in Different Settings
eTable 3. Utility and Cost Parameters in the Model in the Chinese Setting
eTable 4. Yearly Cardiovascular Disease Risk Extracted From Different 10-Year Cardiovascular Risk Prediction Models in the Chinese Setting
eFigure 4. Probability of Cost-effectiveness of Intensive vs Standard Blood Pressure Control in Different Adherence Scenarios in China
eFigure 5. One-way Sensitivity Analysis Presented as a Tornado Diagram for the Chinese Setting
eTable 5. Assumptions in the Model and Relevant Scenario Analyses in the Chinese Setting
eTable 6. Cost-effectiveness of Intensive vs Standard Blood Pressure Control Among Older Patients With Different Stratifications in China
eFigure 6. Probability of Cost-effectiveness of Intensive vs Standard Blood Pressure Control by Age, Sex, and Systolic Blood Pressure at Baseline in China
eTable 7. Utility and Cost Parameters Simulated in the Model for the US Setting
eTable 8. Yearly Cardiovascular Disease Risk Extracted From Different 10-Year Cardiovascular Risk Prediction Models in the US Setting
eFigure 7. Probability of Cost-effectiveness of Intensive vs Standard Blood Pressure Control in Different Adherence Scenarios in the US

© 2023 Liao CT et al. *JAMA Network Open*.

eFigure 8. One-way Sensitivity Analysis Presented as a Tornado Diagram for the US Setting
eTable 9. Assumptions in the Model and Relevant Scenario Analyses in the US Setting
eTable 10. Cost-effectiveness of Intensive vs Standard Blood Pressure Control Among Older Patients With Different Stratifications in the US
eFigure 9. Probability of Cost-effectiveness of Intensive vs Standard Blood Pressure Control by Age, Sex, and Systolic Blood Pressure at Baseline in the US
eTable 11. Utility and Cost Parameters Simulated in the Model for the UK Setting
eFigure 10. Probability of Cost-effectiveness of Intensive vs Standard Blood Pressure Control in Different Adherence Scenarios in the UK
eFigure 11. One-way Sensitivity Analysis Presented as a Tornado Diagram for the UK Setting
eTable 12. Assumptions in the Model and Relevant Scenario Analyses in the UK Setting
eTable 13. Cost-effectiveness of Intensive vs Standard Blood Pressure Control Among Older Patients With Different Stratifications in the UK Setting
eFigure 12. Probability of Cost-effectiveness of Intensive vs Standard Blood Pressure Control by Age, Sex, and Systolic Blood Pressure at Baseline in the UK
eReferences

This supplementary material has been provided by the authors to give readers additional information about their work.

© 2023 Liao CT et al. *JAMA Network Open*.

eMethods. Hypothetical Cohort and Models

Hypothetic cohort

This study simulated 10,000 STEP-eligible patients in the model. The STEP trial enrolled Han patients aged 60-80 with hypertension with systolic blood pressure between 140-190mmHg (mean value around 146mmHg). Patients with systolic blood pressure ≥190mmHg or diastolic blood pressure <60mmHg were excluded. The study excluded patients with the following criteria: 1. secondary hypertension; 2. ischemic and hemorrhagic stroke; 3. hospitalization for myocardial infarction within six months; 4. coronary revascularization within 12 months or planned revascularization in the next 12 months; 5. history of atrial fibrillation or ventricular arrhythmia; 6. New York Heart Association Class III-IV heart failure hospitalization; 7. severe valvular heart disease; 8. hypertrophic and dilated cardiomyopathy, rheumatic heart disease and congenital heart disease; 9. Uncontrolled diabetes; 10. severe liver, kidney or somatic disease; 11. severe congenital impairment or mental disorder; 12. enrolled in other trials.

The characteristics of the hypothetic patients were similar to those in the STEP trial. The patients were 66 years old and had a body mass index of 25.5kg/m^2, systolic blood pressure of 146mmHg, diastolic blood pressure of 82.5mmHg, fasting serum glucose of 6.2mmol/l, total cholesterol of 4.9mmol/l, triglyceride of 1.3mmol/l, high-density lipoprotein cholesterol of 1.3mmol/l, low-density lipoprotein cholesterol of 2.7mmol/l and high Framingham Risk Score ≥ 15%.

Definition of clinical events

This cost-effectiveness analysis simulated the STEP-eligible patients in the model, so the definitions of clinical events were based on the trial's event definitions. Briefly, the first occurrence of stroke included ischemic and hemorrhagic strokes. The strict neurologic examination and images confirmed the diagnosis. Transient ischemic attack and lacunar infarction were not included. Acute coronary syndrome included myocardial infarction and hospitalization for unstable angina. Diagnosis of acute decompensated heart failure required a hospitalization or emergency room visit where infusion therapy was given to release the clinical signs and symptoms due to cardiac decompensation or impaired cardiac pump function. Coronary revascularization included percutaneous coronary intervention and coronary artery bypass graft. Atrial fibrillation was diagnosed using an electrocardiogram with the typical pattern, including absolutely irregular RR intervals and no discernible, distinct P wave. Cardiac death included fatal coronary heart death, fatal stroke, death from heart

© 2023 Liao CT et al. *JAMA Network Open*.

failure and sudden cardiac death. Hypotension was defined as a systolic blood pressure <110mmHg or diastolic blood pressure <50mmHg. Dizziness was reported if the patient had the false feeling of spinning or moving. The definition of syncope was a transient loss of consciousness due to transient global cerebral hypoperfusion and complete spontaneous recovery. The fracture was defined as the destruction of bone integrity or continuity.

Transition probability of clinical events in the model

The model extracted parameters from the STEP trial with the conversion formula of rate and risk (probability): (1) Probability (obtained from the STEP trial) transformed to a rate: [-ln (1-p)]/t. (2) Rate transformed to a probability (yearly transition probability applied in the analyses): 1-exp(-rt), where r is the rate, p is the probability, and t is the time. For example, the stroke probability in the intensive group was 48/4243 within the follow-up period (four years). We firstly converted it to a one-year rate {[-ln (1-48/4243)]/4=0.002844307}, and then transferred it to a one-year probability for a one-year cycle length of the model [1-exp(-0.002844307)= 0.002840265]. Then, the individual CVD risks were distributed from the total CVD risks according to the percentages of each CVD. For example, the given year probability of acute stroke = total CVD risks x [annual $\text{probability}_{\text{acute\_stroke}}$ / (annual $\text{probability}_{\text{acute\_stroke}}$+annual $\text{probability}_{\text{acute\_coronary\_syndrome}}$+annual $\text{probability}_{\text{coronary\_revascularization}}$+annual $\text{probability}_{\text{acute\_heart\_failure}}$+annual $\text{probability}_{\text{acute\_fibrillation}}$+annual $\text{probability}_{\text{cardiovascular\_death}}$)].

10-year cardiovascular risk assessment models

In this study, we extracted the cardiovascular disease risk from the three 10-year risk assessment models, i.e. SCORE2/SCORE2-OP, China-PAR and AHA/ACC Pooled Cohort Equation risk model. We obtained the treatment effects by using the characteristics of the hypothetic patients to estimate the 10-year cardiovascular disease risk. Then we pooled the annual risk according to the characteristics, i.e. male (46.5%) and female (53.5%), and adherence assumption. Accordingly, we obtained dynamic cardiovascular disease risks with different ages as the treatment effects instead of a consistent probability.

We used the SCORE2/SCORE2-OP for base case analyses because the SCORE2-OP provided projections for older patients (to 100 years). However, SCORE2/SCORE2-OP may not fit the countries out of Europe. Thus, the China-PAR model and the AHA/ACC Pooled Cohort Equation were further used for China and the U.S. to consider the uncertainty resulting from different races and countries. The China-PAR

© 2023 Liao CT et al. *JAMA Network Open*.

model only provided estimations for patients aged ≤ 84 years. The AHA/ACC Pooled Cohort Equation was only used for those aged ≤ 79. Therefore, we used extrapolation methods to estimate the out-of-limitation risks and evaluate the uncertainty in further scenario analyses. Taking the China-PAR model as an example, we obtained the function with the best R-squared value by calculating the yearly risks from the original model (66 to 84 years) in the China-PAR model, i.e., $y = 0.0006e^{0.0393x}$, $R^2 = 0.999$ (y, the estimate of yearly CVD risk; x, age). Then, we forward extrapolated the yearly risks to the lifelong period based on the obtained estimates. In the sensitivity analyses, we tested the scenarios with simulation to only 84 years in the China-PAR model and 79 years in the AHA/ACC Pooled Cohort Equation to assess the uncertainty resulting from the extrapolation methods. Besides, although these three models were used in the post-trial period to simulate the different treatment effects, the individual CVD events of the models did not perfectly match the STEP trial. Therefore, we used further scenario analyses with different risk ranges in the model to assess the ICER values.

Adherence

Adherence is crucial for reaching the target in blood-pressure management. In this model, the percentage of the blood pressure reaching the target was assumed to reflect treatment adherence. Patients in the intensive treatment group who did not adhere to their medications reverted to either their baseline, pretrial systolic blood pressure (major) or the standard treatment targets (minor). Those in the standard treatment group all reverted to their baseline. The CVD risk assessment models were used to estimate the treatment effects according to the SBP and other characteristics.

The meta-analysis of M. Iskedjian et al. 2002 showed that the more medications, the lower adherence. The mean numbers of medications in the STEP trial were 1.9 and 1.5 in the intensive and standard groups, and the percentage of blood pressure reaching the target was 76% in both groups at the end of the trial. Therefore, for the base-case analyses, adherence to intensive and standard treatments was assumed to be 70% and 75% based on the target-reaching percentages and the number of antihypertensive medications. In the worst-case scenario, patients in the intensive group were assumed to be non-adherent (0%) to antihypertensive medications immediately after the trial. In contrast, the standard group had perfect adherence (100%) and obtained the treatment effects. Regarding the best-case scenario, the patients in the intensive group adhered to all medications (100%), and the standard group adhered to medications as in the base case. Different adherence changes with time were considered in the scenario analyses.

© 2023 Liao CT et al. *JAMA Network Open*.

Costs

Medical costs came from the published data. We inflated the costs by 3% annually to 2022. If the costs of non-cardiovascular death and cardiovascular death were lacking, cardiovascular events were pooled to estimate the costs of cardiovascular death, and we assumed equal costs for cardiovascular and non-cardiovascular death. We used the purchasing power parity currency instead of the nominal exchange rate to compare the costs between the three countries. The currency was compared between China vs the U.S. (¥4.18=$1) and the U.K. vs the U.S. (£0.688 = $1)

Assumption for repeated cardiovascular events

This model used a Markov model, allowing the simulated individuals to move to the subsequent clinical events according to the given probabilities in each cycle. The probability for the second event would be multiplied by the estimates. For example, the probability of the individual who experienced a stroke (the previous cycle) and then acute heart failure (this cycle) can be obtained by multiplying the probability of stroke and acute heart failure. Usually, repeated CVD risks are higher than the first-time CVD risk. This model assumed that the repeated CVD risks were consistent with the first-time risks, and higher risks for the repeated CVD events were considered in the scenario analyses. The medical costs for this individual in this cycle included baseline healthcare, anti-hypertensive treatments, chronic stroke and acute heart failure. The acute stroke happened in the previous cycle, so the costs were not included in this cycle. The utilities in this cycle were calculated, i.e., $(\text{utility}_{\text{hypertension}} * \text{utility multiplier}_{\text{post\_stroke}} - \text{disutility}_{\text{age}}) - (\text{disutility}_{\text{acute\_heart\_failure}})$. Besides, the costs and utilities were discounted by the given discount rate annually. Finally, lifetime medical costs were calculated by multiplying the number of subjects by the sum of the costs of each health status. Total QALYs were accumulated from the QALY in each cycle, obtained from the utility values associated with each health status multiplied by the proportion of subjects living in that status.

© 2023 Liao CT et al. *JAMA Network Open*.

eFigure 1. Detailed Markov Model Including 2 Treatment Strategies, 6 Health States, Adverse Events, and Cardiovascular Events

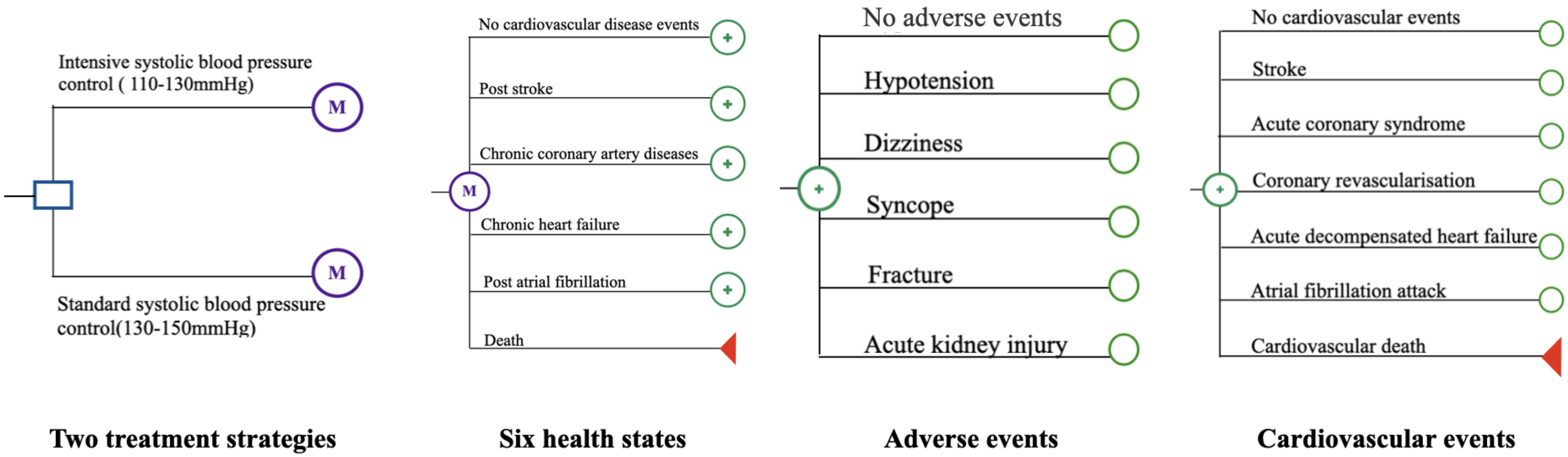

© 2023 Liao CT et al. *JAMA Network Open*.

eFigure 2. Example of the Markov Model Simulation in This Study

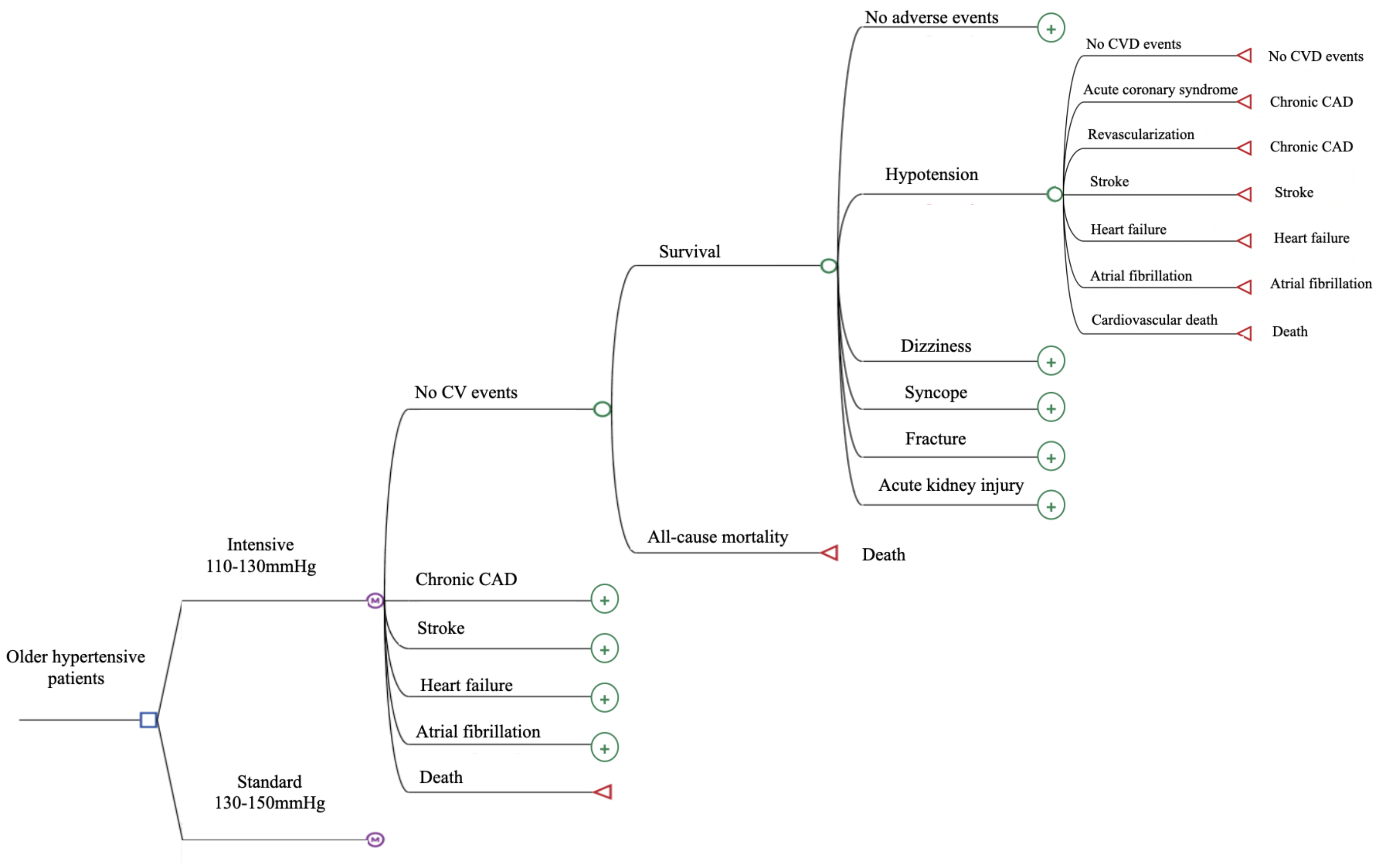

© 2023 Liao CT et al. *JAMA Network Open*.

eFigure 3. Cumulative Incidence of Primary Cardiovascular Outcomes Between Intensive and Standard Blood Pressure Control in Older Patients in the Simulated Model and the STEP Trial

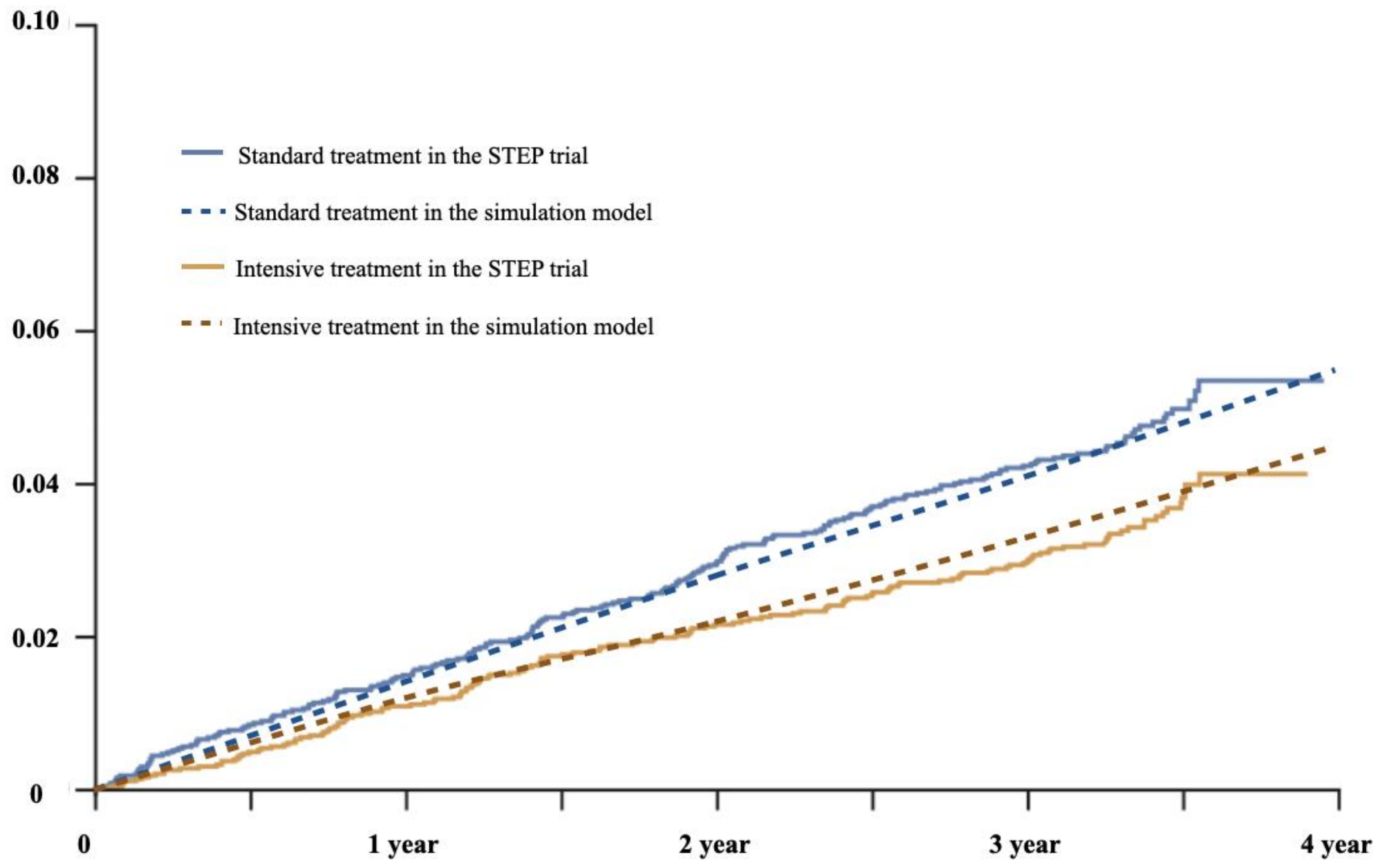

© 2023 Liao CT et al. *JAMA Network Open*.

eTable 1. Comparison of the Percentage of Cardiovascular Events in the Model After the Trial-Period Simulation and in the STEP Trial

	Percentage of patients with intensive treatment in the simulation (%)	Percentage of patients with intensive treatment in the STEP trial (%)	Absolute difference	Percentage of patients with standard treatment in the simulation (%)	Percentage of patients with standard treatment in the STEP trial (%)	Absolute difference
Primary outcomes	3.5	3.5	0	4.1	4.6	0.5
Stroke events	1.0	1.1	0.1	1.3	1.7	0.4
CHD events	1.5	1.8	0.3	2.1	2.6	0.5
Heart failure events	0.1	0.1	0	0.3	0.3	0
Atrial fibrillation	0.5	0.6	0.1	0.4	0.6	0.2
CV death	0.4	0.4	0	0.5	0.6	0.1
All-cause death	1.6	1.6	0	1.4	1.5	0.1

CHD, coronary heart diseases, including patient events with acute coronary syndrome or revascularization

© 2023 Liao CT et al. *JAMA Network Open*.

eTable 2. Cardiovascular Events per 1000 Patients With Intensive and Standard Treatments After Lifetime Simulation in Different Settings

	China			The U.S.			The U.K.		
	Intensive	Standard	Difference	Intensive	Standard	Difference	Intensive	Standard	Difference
Base-case analysis									
Total CV events	2069	2422	353	2691	3016	325	2656	3084	428
CHD events	915	1,130	215	1,213	1,424	211	1,166	1,429	263
Stroke events	710	808	98	930	1,012	82	919	1,039	120
Heart failure events	79	184	105	104	232	128	103	238	135
Atrial fibrillation events	331	265	-66	404	306	-98	426	334	-92
CV death	34	35	1	40	42	2	42	44	2
Non-CV death	966	965	-1	960	958	-2	958	956	-2
Worst-case analysis									
Total CV events	2287	2423	136	2972	2968	-4	2928	3082	154
CHD events	1005	1130	125	1,328	1,403	75	1,277	1,425	148
Stroke events	790	806	16	1,039	995	-44	1,021	1,037	16
Heart failure events	88	185	97	117	228	111	114	238	124
Atrial fibrillation events	367	266	-101	442	301	-141	470	338	-132
CV death	37	36	-1	46	41	-5	46	44	-2
Non-CV death	963	964	1	954	959	5	954	956	2
Best-case analysis									
Total CV events	1974	2418	444	2580	3016	436	2539	3089	550

© 2023 Liao CT et al. *JAMA Network Open*.

eTable 2. Cardiovascular Events per 1000 Patients With Intensive and Standard Treatments After Lifetime Simulation in Different Settings

	China			The U.S.			The U.K.		
	**Intensive**	**Standard**	**Difference**	**Intensive**	**Standard**	**Difference**	**Intensive**	**Standard**	**Difference**
CHD events	876	1128	252	1,168	1,424	256	1,118	1,429	311
Stroke events	675	806	131	887	1,012	125	876	1,039	163
Heart failure events	75	184	109	98	232	134	98	238	140
Atrial fibrillation events	316	265	-51	389	306	-83	407	339	-68
CV death	32	35	3	38	42	4	40	44	4
Non-CV death	968	965	-3	962	958	-4	960	956	-4

CHD, coronary heart diseases, including patient events with acute coronary syndrome or revascularization. CV, cardiovascular

© 2023 Liao CT et al. *JAMA Network Open*.

eTable 3. Utility and Cost Parameters in the Model in the Chinese Setting

	Estimates	Range	Distribution	Reference
Utility				
Hypertension (age and sex dependent)	0.75	0.74–0.77	Uniform	Liu X et al.
Health event multiplier				
Acute coronary syndrome	0.76	0.50-0.87	Beta	Li C et al.
Stroke	0.63	0.26-0.92	Beta	Li C et al.
Post stroke	0.63	0.26-0.92	Beta	Li C et al.
Acute heart failure	0.64	0.43-0.84	Beta	Bress AP et al.
Atrial fibrillation	0.81	0.65-0.97	Beta	AR Harrington et al.
Utility decrements				
Age per year	-0.003	-0.002 - -0.004	Beta	Liu X et al.
Chronic coronary heart diseases	-0.12	-0.06 - -0.33	Beta	Li C et al.
Acute kidney injury	-0.323	-0.26 - -0.38	Beta	Margaret C et al. Applied for 4 weeks
Fracture	-0.343	-0.28 - -0.40	Beta	Margaret C et al. Applied for 12 weeks
Other adverse events	-0.1	-0.08 - -0.13	Beta	Bress AP et al. Applied for 2 weeks
Cardiovascular event costs (US$)				
Acute coronary syndrome	14,061	4,687-42,184	Gamma	Li C et al.
Stroke	6,274	2,091-18,822	Gamma	Li C et al.
Coronary revascularization	4,641	2,320-6,961	Gamma	Li C et al.
Heart failure	3,027	951-8,559	Gamma	Li C et al.
Atrial fibrillation	320	160-780	Gamma	Hu S et al.

© 2023 Liao CT et al. *JAMA Network Open*.

eTable 3. Utility and Cost Parameters in the Model in the Chinese Setting

	Estimates	Range	Distribution	Reference
Annual costs of cardiovascular states (US$)				
Chronic coronary heart diseases	784	246-2,217	Gamma	Li C et al.
Chronic Stroke	2,700	848-7,635	Gamma	Li C et al.
Chronic heart failure	625	196-1,767	Gamma	Li C et al.
Chronic atrial fibrillation	203	101-304	Gamma	Wei H et al
Adverse event costs (US$)				
Hypotension	56	28-85	Gamma	Liu P et al.
Dizziness	56	28-85	Gamma	Assume the same costs as hypotension
Syncope	406	203-609	Gamma	Li YW et al.
Acute kidney injury	4,538	2,269-6,807	Gamma	Fang Y et al.
Fracture	3,567	1,784-5,351	Gamma	Yang Y et al.
Anti-hypertension related medical costs (US$)				
Annual costs for intensive treatment	681	413-1,155	Gamma	Li C et al.
Annual costs for standard treatment	408	258-757	Gamma	Li C et al.
Other related medical costs (US$)				
Background medical costs	800	400-1,200	Gamma	Yang L et al.
Cardiovascular death	5,841	2,920-8,761	Gamma	Assumption
Non cardiovascular death	5,841	2,920-8,761	Gamma	Assumption

All costs were inflated to 2022. Assumption for costs of cardiovascular death was estimated by averaging the costs of acute coronary syndrome, stroke and heart failure. Assumption for costs of non-cardiovascular death was estimated as equal to cardiovascular death

© 2023 Liao CT et al. *JAMA Network Open*.

eTable 4. Yearly Cardiovascular Disease Risk Extracted From Different 10-Year Cardiovascular Risk Prediction Models in the Chinese Setting

	Intensive	Standard	Poor adherence	Intensive	Standard	Poor adherence
STEP trial (66-70 years)	0.008776182	0.01168397	N/A	0.008776182	0.01168397	N/A
10-year cardiovascular risk prediction model	Yearly risk of cardiovascular events calculated from the SCORE2 and SCORE2-OP models			Yearly risk of cardiovascular events calculated from the CHINA-PAR model[a]		
66	0.007404405	0.00819037	0.00952562	0.00741189	0.0091638	0.01076698
67	0.007940292	0.0087301	0.00981757	0.00773313	0.0094902	0.01109817
68	0.008478798	0.00952562	0.01062101	0.00810533	0.0098684	0.01143037
69	0.009019949	0.01062101	0.01143037	0.0084788	0.01029125	0.01170408
70	0.008478798	0.00981757	0.01143037	0.00885353	0.01062101	0.01203812
71	0.009019949	0.01091589	0.01254502	0.00922955	0.01100313	0.01242521
72	0.009817569	0.01172743	0.01367111	0.00960685	0.01133504	0.01276146
73	0.010915891	0.01254502	0.01427835	0.00998545	0.01171965	0.01309874
74	0.012025301	0.01367111	0.01542249	0.01036536	0.01216533	0.01337664
75	0.013146037	0.01480888	0.01712056	0.01080556	0.01255286	0.01376849
76	0.014278347	0.01595861	0.018295	0.01123979	0.0128895	0.01410889
77	0.015422486	0.01774731	0.01948223	0.01168352	0.01327961	0.01445035
78	0.016578717	0.01892856	0.02068253	0.01212124	0.01367111	0.01484606
79	0.018928559	0.02068253	0.02255116	0.01256855	0.0141248	0.01525125
80	0.020122745	0.02255116	0.02436507	0.01307003	0.01451934	0.01564987
81	0.022551162	0.02436507	0.02629879	0.01357381	0.01497657	0.01599618

© 2023 Liao CT et al. *JAMA Network Open*.

eTable 4. Yearly Cardiovascular Disease Risk Extracted From Different 10-Year Cardiovascular Risk Prediction Models in the Chinese Setting

	Intensive	Standard	Poor adherence	Intensive	Standard	Poor adherence
STEP trial (66-70 years)	0.008776182	0.01168397	N/A	0.008776182	0.01168397	N/A
10-year cardiovascular risk prediction model	Yearly risk of cardiovascular events calculated from the SCORE2 and SCORE2-OP models			Yearly risk of cardiovascular events calculated from the CHINA-PAR model[a]		
82	0.023786032	0.02629879	0.02817712	0.01407991	0.01537419	0.01639754
83	0.026298786	0.02887129	0.0301809	0.01458837	0.01583501	0.01674623
84	0.02887129	0.03150663	0.03284889	0.0150992	0.01628967	0.01715853
85	0.031506628	0.03357394	0.03558481	0.01582632	0.01675438	0.01756419
86	0.034942452	0.03632865	0.03773304	0.01644751	0.0180171	0.01823508
87	0.037733038	0.03992532	0.04137814	0.01709309	0.01859368	0.01870979
88	0.041378135	0.04285104	0.04434463	0.01776401	0.0191887	0.01919687
89	0.045046372	0.0473964	0.04822787	0.01846126	0.01980276	0.01969662
90	0.048955902	0.05053877	0.05139545	0.01918588	0.02043648	0.02020939
91	0.053777629	0.05466119	0.05555226	0.01993894	0.02109048	0.0207355
92	0.058031741	0.05975859	0.0606943	0.02072155	0.0217654	0.02127531
93	0.064268612	0.06426861	0.06524592	0.02153489	0.02246192	0.02182917
94	0.069871779	0.07090367	0.07000621	0.02238015	0.02318074	0.02239745
95	0.076889604	0.07593866	0.07593866	0.02325859	0.02392255	0.02298053
96	0.083246691	0.08223506	0.08123336	0.0241715	0.02468811	0.02357878
97	0.091285481	0.08894602	0.08787632	0.02512025	0.02547816	0.02419261

© 2023 Liao CT et al. *JAMA Network Open*.

eTable 4. Yearly Cardiovascular Disease Risk Extracted From Different 10-Year Cardiovascular Risk Prediction Models in the Chinese Setting

	Intensive	Standard	Poor adherence	Intensive	Standard	Poor adherence
STEP trial (66-70 years)	0.008776182	0.01168397	N/A	0.008776182	0.01168397	N/A
10-year cardiovascular risk prediction model	Yearly risk of cardiovascular events calculated from the SCORE2 and SCORE2-OP models			Yearly risk of cardiovascular events calculated from the CHINA-PAR model[a]		
98	0.098648488	0.09630774	0.09385024	0.02610624	0.02629349	0.02482242
99	0.108082722	0.10406394	0.10282158	0.02713093	0.02713492	0.02546862
100+	0.116859843	0.11247605	0.10979106	0.02819584	0.02800328	0.02613165

[a] China-PAR risk model provides 10-years cardiovascular risks for people aged younger than 85 years. The yearly risk for those aged more than 85 years was extrapolated by the function based on the yearly risk from 66 to 85 years (grey column).

The risks were calculated from the risk of males and females according to the sample percentage in the STEP trial.

© 2023 Liao CT et al. *JAMA Network Open*.

eFigure 4. Probability of Cost-effectiveness of Intensive vs Standard Blood Pressure Control in Different Adherence Scenarios in China.

The curve presents the results after running 1000 simulations with random draws for all input parameters to capture joint uncertainty, and the probabilities of cost-effectiveness of intensive treatments changed with different willingness-to-pay (WTP) thresholds (costs in US$ per quality-adjusted life-year gained).

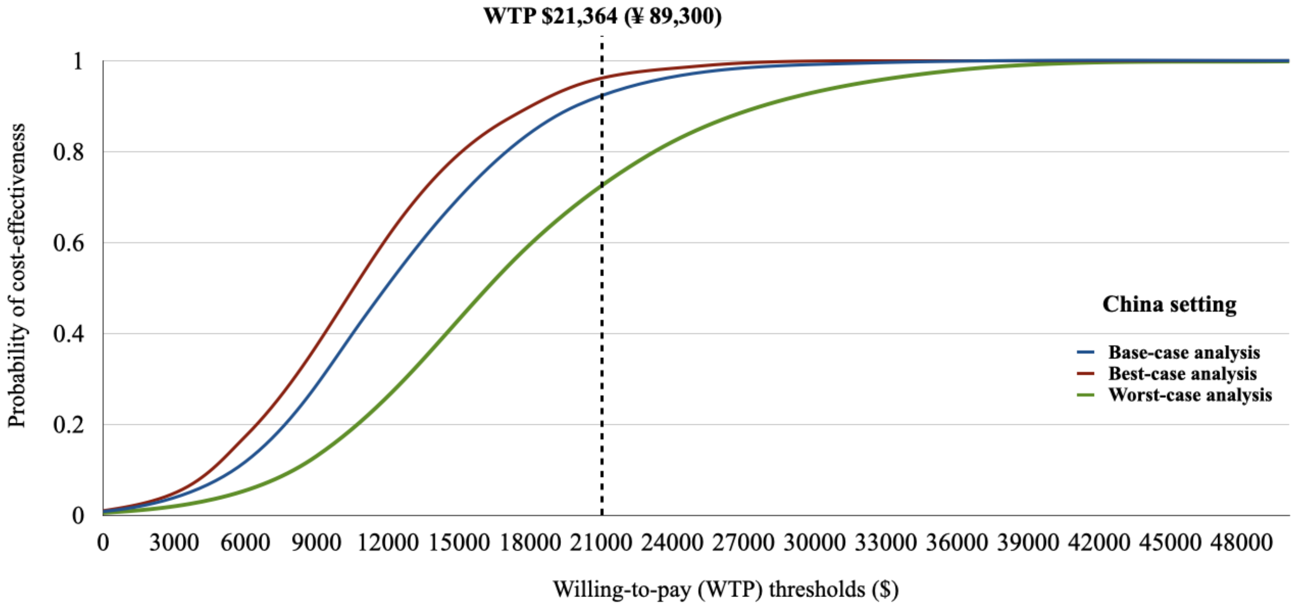

© 2023 Liao CT et al. *JAMA Network Open*.

eFigure 5. One-way Sensitivity Analysis Presented as a Tornado Diagram for the Chinese Setting

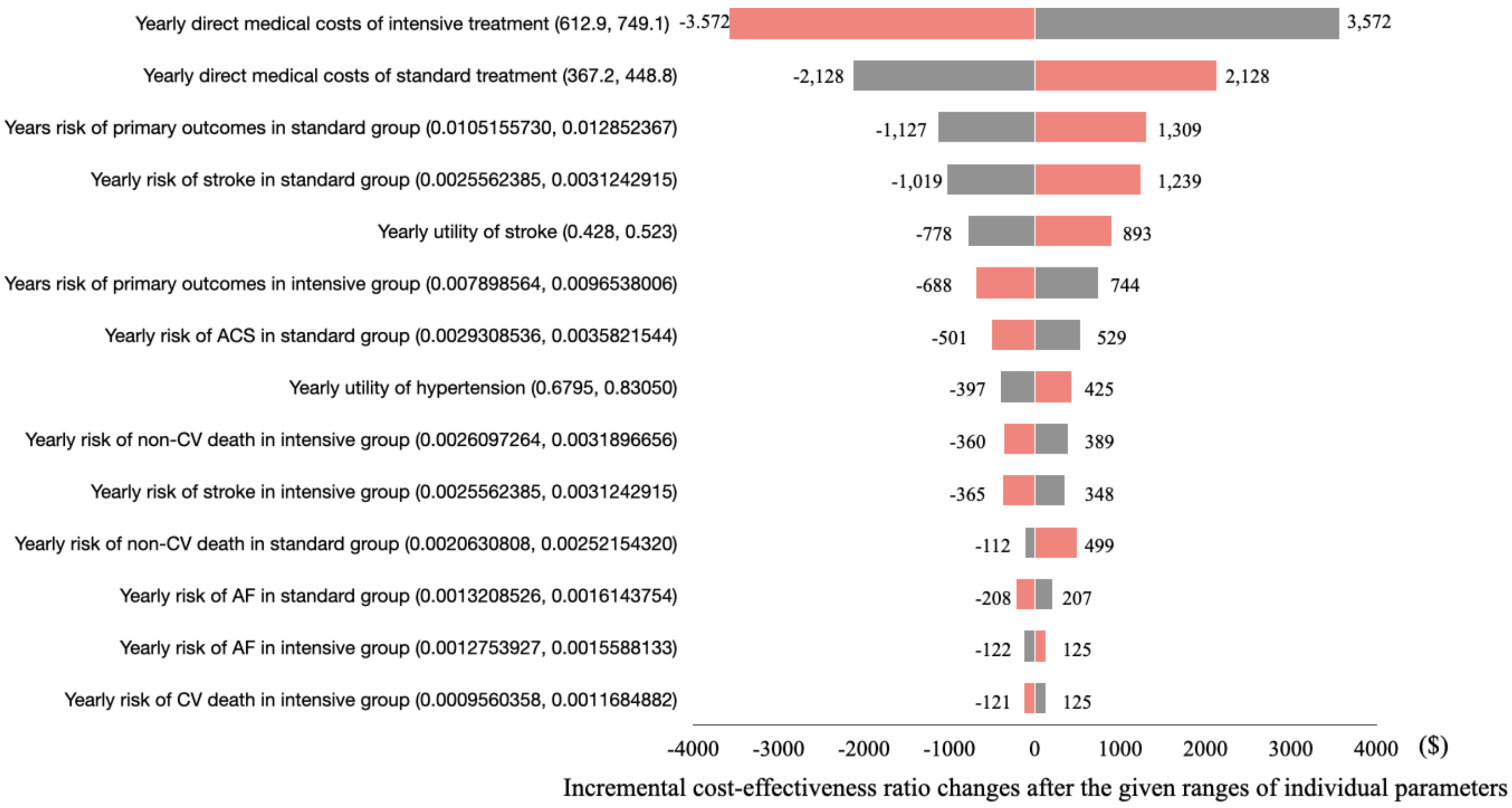

© 2023 Liao CT et al. *JAMA Network Open*.

Supplementary materials of scenario and subgroup analyses in China

eTable 5. Assumptions in the Model and Relevant Scenario Analyses in the Chinese Setting

Assumption	Original assumption	Scenario analyses	ICER value	Possibility of cost-effectiveness at the willingness-to-pay threshold of ¥89,300/QALY	Possibility of cost-effectiveness at the willingness-to-pay threshold of ¥267,900/QALY
Adherence	For base case, adherence in post-trial period for intensive versus standard control: 70% versus 75%	1. Adherence decreased to 60% for both groups	¥52,016	92.6%	100%
		2. Adherence loss rates after trial period for intensive and standard treatment were assumed at 5% and 3% per year	¥50,728	92.8%	100%
	For worst-case, adherence in post-trial period for intensive versus standard control: 0% versus 100%	3. Adherence decreased to 60% for standard control group	¥67,666	75%	100%
	For best-case, adherence in post-trial period for intensive versus standard control: 100% versus 75%	4. Adherence decreased to 60% for standard control group	¥52,384	91.1%	100%

© 2023 Liao CT et al. *JAMA Network Open*.

eTable 5. Assumptions in the Model and Relevant Scenario Analyses in the Chinese Setting

Assumption	Original assumption	Scenario analyses	ICER value	Possibility of cost-effectiveness at the willingness-to-pay threshold of ¥89,300/QALY	Possibility of cost-effectiveness at the willingness-to-pay threshold of ¥267,900/QALY
**Supplementary Table S5**. Assumptions in the model and relevant scenario analyses in the Chinese setting					
Assumption	Original assumption	Scenario analyses	ICER value	Possibility of cost-effectiveness at the willingness-to-pay threshold of ¥89,300/QALY	Possibility of cost-effectiveness at the willingness-to-pay threshold of ¥267,900/QALY
Costs	Intensive treatment annual costs ¥2,847 (US$681) and standard treatment annual costs ¥1,705 ($408)	5. Doubled the intensive treatment annual costs ¥5693(US$1,362)	¥199,658	0.1%	84.2%
		6. 1.5 times the intensive treatment annual costs	¥124,995	12.1%	99.3%
		7. Intensive treatment annual costs only ¥4,180 (US$100) higher than standard treatment	¥12,402	100%	100%

© 2023 Liao CT et al. *JAMA Network Open*.

eTable 5. Assumptions in the Model and Relevant Scenario Analyses in the Chinese Setting

Assumption	Original assumption	Scenario analyses	ICER value	Possibility of cost-effectiveness at the willingness-to-pay threshold of ¥89,300/QALY	Possibility of cost-effectiveness at the willingness-to-pay threshold of ¥267,900/QALY
		8. Intensive treatment annual costs equal to standard treatment	-¥2,279	Cost-saving	Cost-saving
		9. Intensive treatment annual costs decreased to ¥1,887 (US$451.4)	¥0	100% - cost saving	100% - cost saving
**Supplementary Table S5**. Assumptions in the model and relevant scenario analyses in the Chinese setting					
Assumption	Original assumption	Scenario analyses	ICER value	Possibility of cost-effectiveness at the willingness-to-pay threshold of ¥89,300/QALY	Possibility of cost-effectiveness at the willingness-to-pay threshold of ¥267,900/QALY
Costs	CHD yearly costs ¥ 32,77 (US$784)	10. Double the costs ¥6,554 (US$1568)	¥48,442	93.5%	100%
		11. Half the costs ¥16,39 (US$392)	¥51,280	91.9%	100%

© 2023 Liao CT et al. *JAMA Network Open*.

eTable 5. Assumptions in the Model and Relevant Scenario Analyses in the Chinese Setting

Assumption	Original assumption	Scenario analyses	ICER value	Possibility of cost-effectiveness at the willingness-to-pay threshold of ¥89,300/QALY	Possibility of cost-effectiveness at the willingness-to-pay threshold of ¥267,900/QALY
	Acute stroke costs ¥26,225 (US$6,274)	12. Double the costs. ¥52,451 (US$12,548)	¥49,437	91.6%	100%
		13. Half the costs ¥13,113 (US$3,137)	¥50,787	90.9%	100%
	Acute coronary syndrome costs ¥58,775 (US$14,061)	14. Double the costs. ¥117,550 (US$28,122)	¥47,865	92.2%	100%
		15. Half the costs ¥29,385 (US$7,030)	¥51,569	90.2%	100%
	Chronic stroke annual costs ¥11,286 (US$2,700)	16. Double the costs. ¥22,752 ($5,400)	¥47,334	99.8%	100%
		17. Half the costs. ¥ 5,643 (US$1,350)	¥51,836	89.9%	100%
**Supplementary Table S5**. Assumptions in the model and relevant scenario analyses in the Chinese setting					
Assumption	Original assumption	Scenario analyses	ICER value	Possibility of cost-effectiveness at the willingness-	Possibility of cost-effectiveness at the willingness-to-pay

© 2023 Liao CT et al. *JAMA Network Open*.

eTable 5. Assumptions in the Model and Relevant Scenario Analyses in the Chinese Setting

Assumption	Original assumption	Scenario analyses	ICER value	Possibility of cost-effectiveness at the willingness-to-pay threshold of ¥89,300/QALY	Possibility of cost-effectiveness at the willingness-to-pay threshold of ¥267,900/QALY
				to-pay threshold of ¥89,300/QALY	threshold of ¥267,900/QALY
Hazard ratio of primary outcomes during trial period	Hazard ratio: 0.74	18. Using the hazard ratio value (0.68) for patients aged 75 years and over in the SPRINT trial	¥47,606	92.5%	100%
		19. Intensive control had the same hazard as standard control during trial period	¥61,697	82.7%	100%
10-year CVD risk prediction model	Based on SCORE2/SCORE2-OP 10-year risk prediction	20. Increased by 50%	¥36,274	100%	100%
		21. Decreased by 50%	¥80,615	59.5%	99.6%
	Based on China-PAR 10-year cardiovascular risk prediction	22. Lifetime	¥69,777	73.4%	99.7%
		23. Simulated to 85 years	¥70.,926	69.4%	99.6%
Overall mortality	Based on the lifetable from China statistics	24. Increased by 50% during post-trial period	¥61,985	80%	100%

© 2023 Liao CT et al. *JAMA Network Open*.

eTable 5. Assumptions in the Model and Relevant Scenario Analyses in the Chinese Setting

Assumption	Original assumption	Scenario analyses	ICER value	Possibility of cost-effectiveness at the willingness-to-pay threshold of ¥89,300/QALY	Possibility of cost-effectiveness at the willingness-to-pay threshold of ¥267,900/QALY
		25. Decreased by 50% during post-trial period	¥36,658	100%	100%

Supplementary Table S5. Assumptions in the model and relevant scenario analyses in the Chinese setting

Assumption	Original assumption	Scenario analyses	ICER value	Possibility of cost-effectiveness at the willingness-to-pay threshold of ¥89,300/QALY	Possibility of cost-effectiveness at the willingness-to-pay threshold of ¥267,900/QALY
Adverse event risk	Based on the STEP trial	26. Intensive treatment doubled the risk of adverse events	¥54,938	89.2%	100%
		27. Risk of adverse events doubled in both groups	¥49,671	93.9%	100%

© 2023 Liao CT et al. *JAMA Network Open*.

eTable 5. Assumptions in the Model and Relevant Scenario Analyses in the Chinese Setting

Assumption	Original assumption	Scenario analyses	ICER value	Possibility of cost-effectiveness at the willingness-to-pay threshold of ¥89,300/QALY	Possibility of cost-effectiveness at the willingness-to-pay threshold of ¥267,900/QALY
Repeated CVD risk	Based on the STEP trial	28. Intensive treatment doubled the risk of recurrent CVD	¥58,549	85.9%	100%
		29. Risk of recurrent CVD doubled in both groups	¥46,373	96%	100%
Discount rate	Original value: 3%	30. Discount rate at 0	¥39,351	100%	100%
		31. Discount rate at 5%	¥59,281	79.7%	100%
**Supplementary Table S5**. Assumptions in the model and relevant scenario analyses in the Chinese setting					
Assumption	Original assumption	Scenario analyses	ICER value	Possibility of cost-effectiveness at the willingness-to-pay threshold of ¥89,300/QALY	Possibility of cost-effectiveness at the willingness-to-pay threshold of ¥267,900/QALY

© 2023 Liao CT et al. *JAMA Network Open*.

eTable 5. Assumptions in the Model and Relevant Scenario Analyses in the Chinese Setting

Assumption	Original assumption	Scenario analyses	ICER value	Possibility of cost-effectiveness at the willingness-to-pay threshold of ¥89,300/QALY	Possibility of cost-effectiveness at the willingness-to-pay threshold of ¥267,900/QALY
Cycle length	Lifetime	32. Only trial period: four years	¥276,829	20.2%	45.1%
		33. 10 years	¥128,247	32%	81.5%
		34. 11 years (close to the life expectancy of 77 years in China)	¥93,247	46.2%	94.9%
		35. 20 years	¥71,123	76.5%	99.4%
		36. 30 years	¥60,058	81.7%	100%

© 2023 Liao CT et al. *JAMA Network Open*.

eTable 6. Cost-effectiveness of Intensive vs Standard Blood Pressure Control Among Older Patients With Different Stratifications in China

Variables	Subgroup	Primary-outcome yearly hazard ratio	ICER	PSA at different WTP thresholds	
			Cost per QALY gained	¥89,300/QALY	¥267,900/QALY
Age	60-69 years	0.75±0.26	¥51,432	92.1%	100%
	70-80 years	0.74±0.37	¥51,833	83.2%	100%
Sex	Men	0.71±0.30	¥56,194	87.8%	100%
	Women	0.80±0.31	¥74,406	70.1%	100%
Systolic blood pressure	≤138 mmHg	0.71±0.39	¥49,257	94.7%	100%
	139–151 mmHg	0.95±0.36	¥59,564	84.6%	100%
	≥152 mmHg	0.62±0.37	¥46,221	95.9%	100%
Previous diabetes	No	0.74±0.25	¥51,709	92.7%	100%
	Yes	0.78±0.42	¥52,267	90.9%	100%
Blood pressure measurement	App management	0.78±0.28	¥52,267	93.1%	100%
	Usual management	0.72±0.33	¥49,393	94.8%	100%

ICER, incremental cost-effectiveness ratio; QALY, quality-adjusted life-year; PSA, probabilistic sensitivity analyses

© 2023 Liao CT et al. *JAMA Network Open*.

eFigure 6. Probability of Cost-effectiveness of Intensive vs Standard Blood Pressure Control by Age, Sex, and Systolic Blood Pressure at Baseline in China

A. Subgroups aged 60-69 and 70-80 years in China.

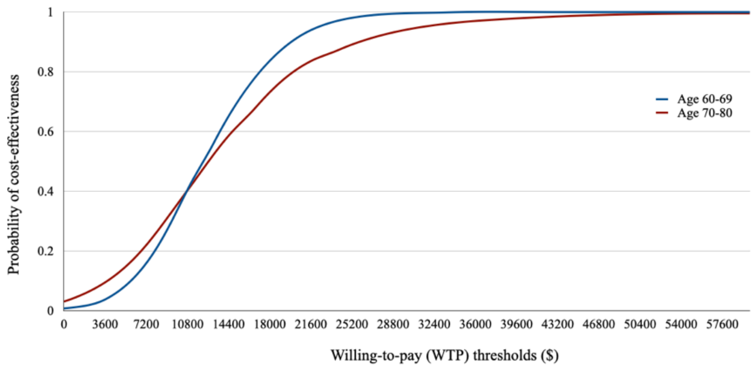

B. Male and female subgroups in China.

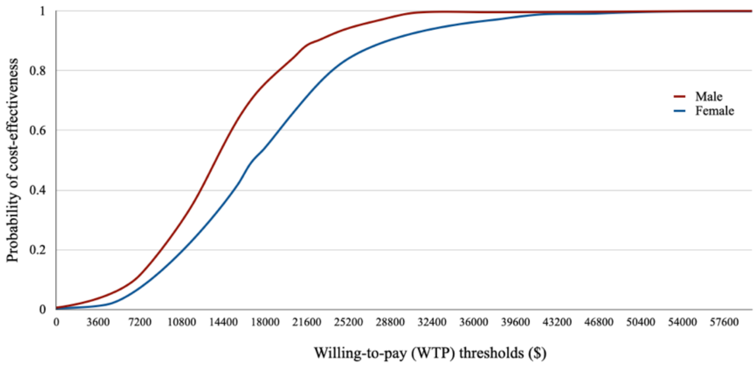

C. Different systolic pressure subgroups at baseline in China.

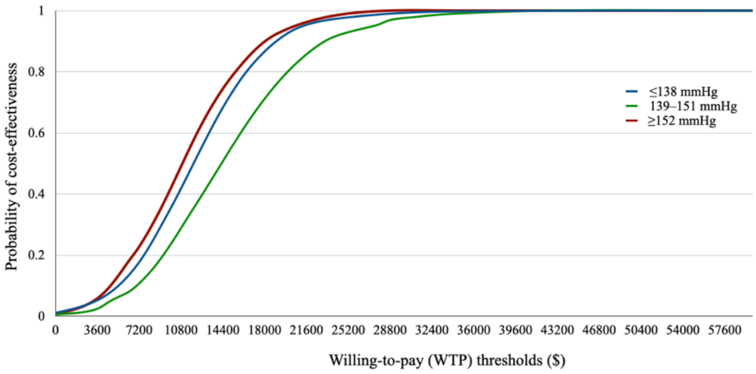

© 2023 Liao CT et al. *JAMA Network Open*.

eTable 7. Utility and Cost Parameters Simulated in the Model for the US Setting

	Estimates	Range	Distribution	Reference
Utility				
Hypertension (age and sex dependent)	0.83	0.75-0.85	Uniform	Fryback DG et al. and Stein JD et al.
Health event multiplier				
Acute coronary syndrome	0.70	0.54-0.86	Beta	Bress AP et al.
Stroke	0.65	0.47-0.83	Beta	Bress AP et al.
Post stroke	0.65	0.47-0.83	Beta	Bress AP et al.
Acute heart failure	0.64	0.44-0.84	Beta	Bress AP et al.
Atrial fibrillation	0.81	0.68-0.94	Beta	Harrington AR et al.
Utility decrements				
Age per year	-0.003	-0.002 - -0.004	Beta	Fryback DG et al. and Lie X. et al.
Chronic CHD	-0.10	-0.08 - -0.13	Beta	Bress AP et al. Applied for 4 weeks
Acute kidney injury	-0.323	-0.26 - -0.38	Beta	Margaret C et al. Applied for 4 weeks
Fracture	-0.343	-0.28 - -0.40	Beta	Margaret C et al. Applied for 12 weeks
Other adverse events	-0.10	-0.08 - -0.13	Beta	Fryback DG et al. Applied for 4 weeks
Cardiovascular event costs ($)				
Acute coronary syndrome	19,982	9,941-29,822	Gamma	Bress AP et al.
Stroke	34,648	17,324-51,973	Gamma	Bress AP et al.
Coronary revascularization	40,555	20,277-60,832	Gamma	Nagle PC et al.
Heart failure	11,845	5,923-17,768	Gamma	Bress AP et al.
Atrial fibrillation	2,319	1,159-3,478	Gamma	Harrington AR

© 2023 Liao CT et al. *JAMA Network Open*.

eTable 7. Utility and Cost Parameters Simulated in the Model for the US Setting

	Estimates	Range	Distribution	Reference
Annual costs of cardiovascular states ($)				
Chronic coronary heart diseases	6,010	3,005-9,015	Gamma	Bress AP et al.
Chronic Stroke	12,142	6,071-18,213	Gamma	Bress AP et al.
Chronic heart failure	7,378	3,689-11,066	Gamma	Bress AP et al.
Chronic atrial fibrillation	3,478	1,739-5,217	Gamma	Harrington AR
Adverse event costs ($)				
Hypotension	8,500	4,249-12,748	Gamma	Saber Tehrani AS et al.
Dizziness	1,063	532-1,595	Gamma	Saber Tehrani AS et al.
Syncope	7,884	3,942-11,826	Gamma	Saber Tehrani AS et al.
Acute kidney injury	11,433	5,716-17,149	Gamma	Saber Tehrani AS et al.
Fracture	19,547	9,774-29,321	Gamma	Bonafede M et al.
Anti-hypertension related medical costs ($)				
Annual costs for intensive treatment	1,292	646-1,938	Gamma	Saber Tehrani AS et al.
Annual costs for standard treatment	629	314-943	Gamma	Saber Tehrani AS et al.
Other related medical costs ($)				
Background medical costs	12,095	6,047-18,142	Gamma	https://www.pgpf.org/blog/2022/02/why-are-americans-paying-more-for-healthcare Centers for Medicare and Medicaid Services, National Health Expenditure Data, December 2021

© 2023 Liao CT et al. *JAMA Network Open*.

eTable 7. Utility and Cost Parameters Simulated in the Model for the US Setting

	Estimates	Range	Distribution	Reference
Cardiovascular death	19,890	9,945-29,834	Gamma	Saber Tehrani AS et al.
Non cardiovascular death	8,445	4,223-12,668	Gamma	French EB et al.

All costs were inflated to 2022.

© 2023 Liao CT et al. *JAMA Network Open*.

eTable 8. Yearly Cardiovascular Disease Risk Extracted From Different 10-Year Cardiovascular Risk Prediction Models in the US Setting

	Intensive	Standard	Poor adherence	Intensive	Standard	Poor adherence
STEP trial (66-70 years)	0.008776182	0.01168397	N/A	0.008776182	0.01168397	N/A
10-year cardiovascular risk prediction model	Yearly risk of cardiovascular events calculated from the SCORE2 and SCORE2-OP models			Yearly risk of cardiovascular events calculated from the AHA/ACC Pooled Cohort Equation model [a]		
66	0.007404405	0.00819037	0.00952562	0.01192889	0.014148103	0.01646719
67	0.007940292	0.0087301	0.00981757	0.01304076	0.015451243	0.01809681
68	0.008478798	0.00952562	0.01062101	0.01427778	0.016894756	0.01975115
69	0.009019949	0.01062101	0.01143037	0.01552893	0.018467533	0.0216177
70	0.008478798	0.00981757	0.01143037	0.01709019	0.020135954	0.02358317
71	0.009019949	0.01091589	0.01254502	0.01861902	0.022074566	0.0257789
72	0.009817569	0.01172743	0.01367111	0.02034571	0.02411502	0.02814932
73	0.010915891	0.01254502	0.01427835	0.02223973	0.026389705	0.03077612
74	0.012025301	0.01367111	0.01542249	0.02428332	0.028782811	0.03361406
75	0.013146037	0.01480888	0.01712056	0.02650235	0.03143462	0.03667873
76	0.014278347	0.01595861	0.018295	0.02903738	0.034299816	0.04012247
77	0.015422486	0.01774731	0.01948223	0.0317049	0.037459848	0.04385167
78	0.016578717	0.01892856	0.02068253	0.0345136	0.041017444	0.04787195
79	0.018928559	0.02068253	0.02255116	0.03775566	0.044778711	0.05229969
80	0.020122745	0.02255116	0.02436507	0.03592531	0.047900415	0.05987552
81	0.022551162	0.02436507	0.02629879	0.03925356	0.052338078	0.0654226
82	0.023786032	0.02629879	0.02817712	0.04289015	0.057186861	0.07148358

© 2023 Liao CT et al. *JAMA Network Open*.

eTable 8. Yearly Cardiovascular Disease Risk Extracted From Different 10-Year Cardiovascular Risk Prediction Models in the US Setting

	Intensive	Standard	Poor adherence	Intensive	Standard	Poor adherence
STEP trial (66-70 years)	0.008776182	0.01168397	N/A	0.008776182	0.01168397	N/A
10-year cardiovascular risk prediction model	Yearly risk of cardiovascular events calculated from the SCORE2 and SCORE2-OP models			Yearly risk of cardiovascular events calculated from the AHA/ACC Pooled Cohort Equation model [a]		
83	0.026298786	0.02887129	0.0301809	0.04686364	0.062484853	0.07810607
84	0.02887129	0.03150663	0.03284889	0.05120525	0.068273669	0.08534209
85	0.031506628	0.03357394	0.03558481	0.05594909	0.074598782	0.09324848
86	0.034942452	0.03632865	0.03773304	0.06113241	0.081509875	0.10188734
87	0.037733038	0.03992532	0.04137814	0.06679593	0.089061236	0.11132654
88	0.041378135	0.04285104	0.04434463	0.07298414	0.097312181	0.12164023
89	0.045046372	0.0473964	0.04822787	0.07974564	0.106327524	0.1329094
90	0.048955902	0.05053877	0.05139545	0.08713356	0.116178079	0.1452226
91	0.053777629	0.05466119	0.05555226	0.09520592	0.126941224	0.15867653
92	0.058031741	0.05975859	0.0606943	0.10402613	0.138701505	0.17337688
93	0.064268612	0.06426861	0.06524592	0.11366347	0.151551299	0.18943912
94	0.069871779	0.07090367	0.07000621	0.12419366	0.165591544	0.20698943
95	0.076889604	0.07593866	0.07593866	0.13569939	0.180932526	0.22616566
96	0.083246691	0.08223506	0.08123336	0.14827106	0.19769475	0.24711844
97	0.091285481	0.08894602	0.08787632	0.16200741	0.216009886	0.27001236
98	0.098648488	0.09630774	0.09385024	0.17701635	0.2360218	0.29502725
99	0.108082722	0.10406394	0.10282158	0.19341577	0.257887689	0.32235961

© 2023 Liao CT et al. *JAMA Network Open*.

eTable 8. Yearly Cardiovascular Disease Risk Extracted From Different 10-Year Cardiovascular Risk Prediction Models in the US Setting

	Intensive	Standard	Poor adherence	Intensive	Standard	Poor adherence
STEP trial (66-70 years)	0.008776182	0.01168397	N/A	0.008776182	0.01168397	N/A
10-year cardiovascular risk prediction model	Yearly risk of cardiovascular events calculated from the SCORE2 and SCORE2-OP models			Yearly risk of cardiovascular events calculated from the AHA/ACC Pooled Cohort Equation model [a]		
100+	0.116859843	0.11247605	0.10979106	0.21133448	0.28177931	0.35222414

[a] AHA/ACC Pooled Cohort Equation risk model provides 10-year cardiovascular risks for people aged younger than 80 years. The yearly risk for those aged more than 80 years was extrapolated by the function based on the yearly risk from 66 to 80 years (grey column).

© 2023 Liao CT et al. *JAMA Network Open*.

eFigure 7. Probability of Cost-effectiveness of Intensive vs Standard Blood Pressure Control in Different Adherence Scenarios in the US

The curve presents the results after running 1000 simulations with random draws for all input parameters to capture joint uncertainty, and the probabilities of cost-effectiveness of intensive treatments changed with different willingness-to-pay (WTP) thresholds (costs in US$ per quality-adjusted life-year gained).

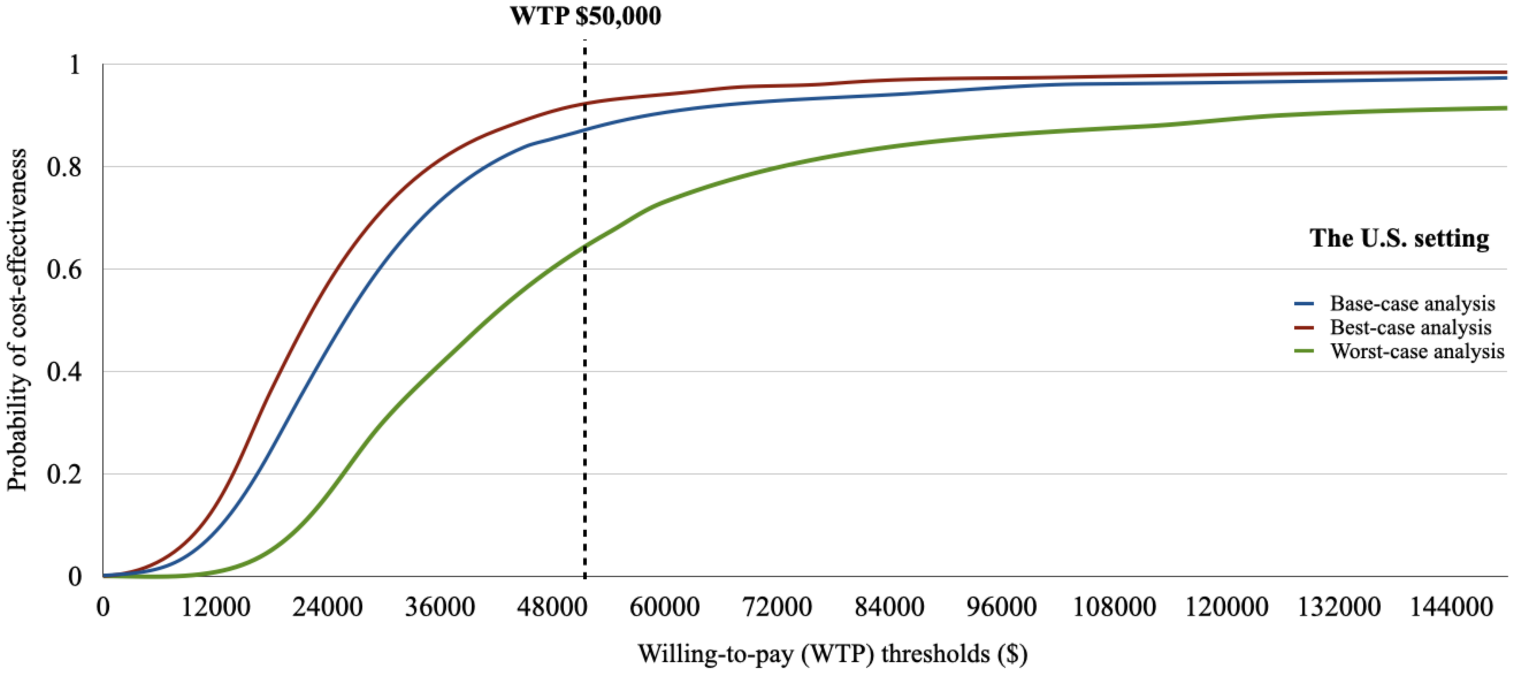

© 2023 Liao CT et al. *JAMA Network Open*.

eFigure 8. One-way Sensitivity Analysis Presented as a Tornado Diagram for the US Setting

ICER, incremental cost-effectiveness ratio.

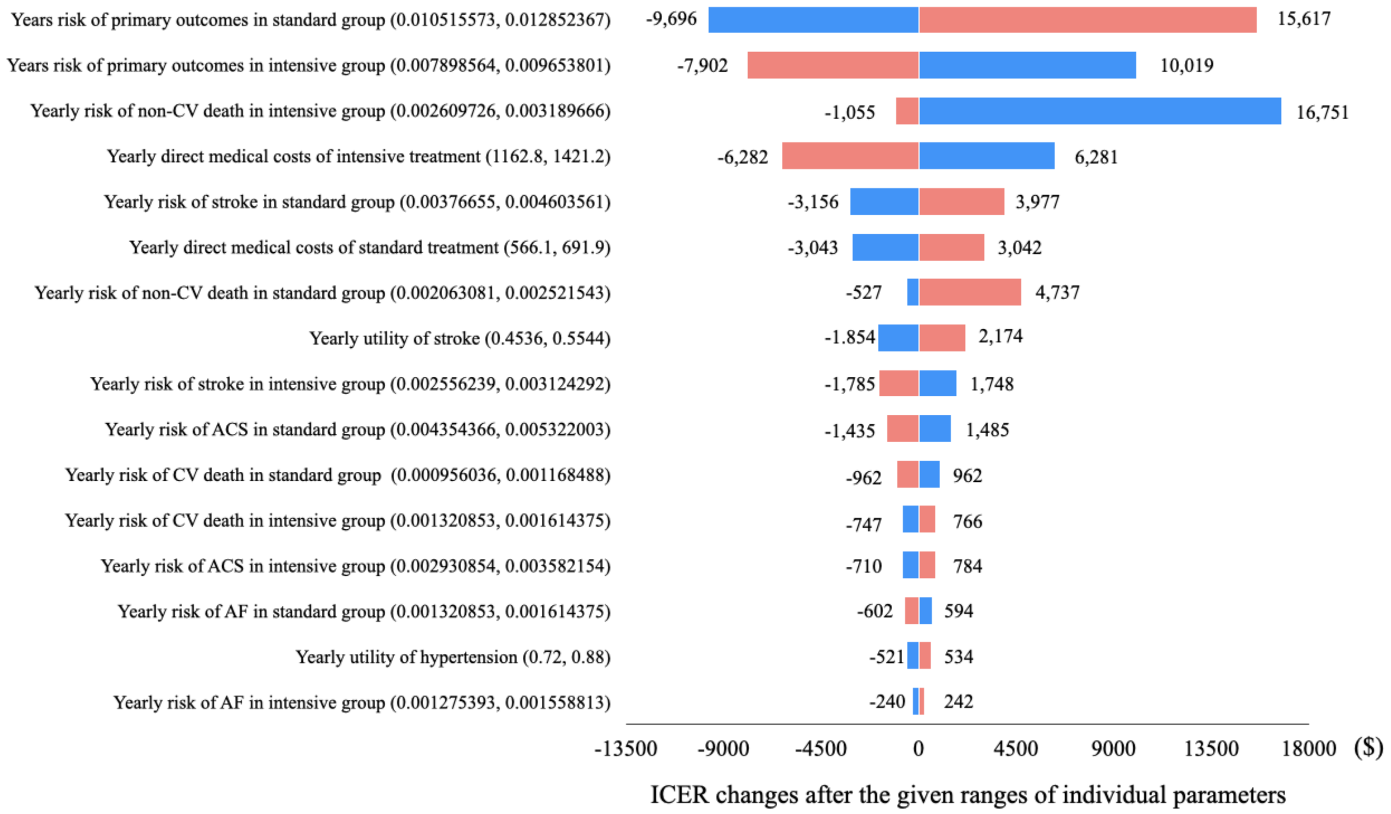

© 2023 Liao CT et al. *JAMA Network Open*.

Supplementary materials for scenario and subgroup analyses in the U.S.

eTable 9. Assumptions in the Model and Relevant Scenario Analyses in the US Setting

Assumption	Original assumption	Scenario analyses	ICER value	Possibility of cost-effectiveness at the willingness-to-pay threshold of $50,000/QALY	Possibility of cost-effectiveness at the willingness-to-pay threshold of $100,000/QALY
Adherence	For base case, adherence in post-trial period for intensive versus standard control: 70% versus 75%	1. Adherence decreased to 60% for both groups	$24,667	87.7%	92.8%
		2. Adherence loss rates after trial period for intensive and standard treatment were assumed at 5% and 3% per year	$26,705	83.9%	90.8%
	For worst-case, adherence in post-trial period for intensive versus standard control: 0% versus 100%	3. Adherence decreased to 60% for standard control group	$35,120	74.8%	84.7%
	For best-case, adherence in post-trial period for intensive versus standard control: 100% versus 75%	4. Adherence decreased to 60% for standard control group	$25,908	86.1%	91.3%

© 2023 Liao CT et al. *JAMA Network Open*.

eTable 9. Assumptions in the Model and Relevant Scenario Analyses in the US Setting

Assumption	Original assumption	Scenario analyses	ICER value	Possibility of cost-effectiveness at the willingness-to-pay threshold of $50,000/QALY	Possibility of cost-effectiveness at the willingness-to-pay threshold of $100,000/QALY
**Supplementary Table S9.** Assumptions in the model and relevant scenario analyses in the U.S. setting					
Assumption	Original assumption	Scenario analyses	ICER value	Possibility of cost-effectiveness at the willingness-to-pay threshold of $50,000/QALY	Possibility of cost-effectiveness at the willingness-to-pay threshold of $100,000/QALY
Costs	Intensive treatment annual costs ($1,292) and standard treatment annual costs ($629)	5. Doubled the intensive treatment annual costs ($2,584)	$87,299	5.6%	63.8%
		6. 1.5 times the intensive treatment annual costs	$56,036	36.2%	79.5%

© 2023 Liao CT et al. *JAMA Network Open*.

eTable 9. Assumptions in the Model and Relevant Scenario Analyses in the US Setting

Assumption	Original assumption	Scenario analyses	ICER value	Possibility of cost-effectiveness at the willingness-to-pay threshold of $50,000/QALY	Possibility of cost-effectiveness at the willingness-to-pay threshold of $100,000/QALY
		7. Intensive treatment annual costs 1.5 times the standard treatment	$7,909	96.9%	100%
		8. Intensive treatment annual costs equal to standard treatment	-$7,313	Cost-saving	Cost-saving
		9. Intensive treatment annual costs decreased to $780	$0	100% - cost-saving	100% - cost-saving

Supplementary Table S9. Assumptions in the model and relevant scenario analyses in the U.S. setting

Assumption	Original assumption	Scenario analyses	ICER value	Possibility of cost-effectiveness at the willingness-to-pay threshold of	Possibility of cost-effectiveness at the willingness-to-pay threshold of

© 2023 Liao CT et al. *JAMA Network Open*.

eTable 9. Assumptions in the Model and Relevant Scenario Analyses in the US Setting

Assumption	Original assumption	Scenario analyses	ICER value	Possibility of cost-effectiveness at the willingness-to-pay threshold of $50,000/QALY	Possibility of cost-effectiveness at the willingness-to-pay threshold of $100,000/QALY
				$50,000/QALY	$100,000/QALY
Costs	CHD yearly costs ($6,010)	10. Double the costs ($12,020)	$22,038	90.5%	95%
		11. Half the costs ($3,005)	$26,142	85.5%	92.2%
	Acute stroke costs ($34,648)	12. Double the costs ($69,296)	$23,872	89.8%	94.3%
		13. Half the costs ($17,324)	$25,223	84.1%	93.1%
	Acute coronary syndrome costs ($19,882)	14. Double the costs. ($39,764)	$24,130	89.3%	93.5%
		15. Half the costs ($9,941)	$25,094	86.1%	93.2%
	Chronic stroke annual costs ($12,142)	16. Double the costs ($24,284)	$22,616	87.4%	94.8%
		17. Half the costs ($6.071)	$25,851	85.1%	92.9%
Hazard ratio of primary outcomes during trial	Hazard ratio: 0.74	18. Using the hazard ratio value (0.68), which was for patients aged 75 years and more in the SPRINT trial	$23,041	88.3%	94.5%

© 2023 Liao CT et al. *JAMA Network Open*.

eTable 9. Assumptions in the Model and Relevant Scenario Analyses in the US Setting

Assumption	Original assumption	Scenario analyses	ICER value	Possibility of cost-effectiveness at the willingness-to-pay threshold of $50,000/QALY	Possibility of cost-effectiveness at the willingness-to-pay threshold of $100,000/QALY
period		19. Intensive control had the same hazard as standard control during trial period	$31,927	75.8%	96.1%

Supplementary Table S9. Assumptions in the model and relevant scenario analyses in the U.S. setting

Assumption	Original assumption	Scenario analyses	ICER value	Possibility of cost-effectiveness at the willingness-to-pay threshold of $50,000/QALY	Possibility of cost-effectiveness at the willingness-to-pay threshold of $100,000/QALY
10-year CVD risk prediction model	Based on SCORE2/SCORE2-OP 10-year risk prediction	20. Increased by 50%	$17,694	97.5%	100%
		21. Decreased by 50%	$40,116	60.1%	84.6%
	Based on AHA/ACC Pooled Cohort Equation risk prediction	22. Lifetime	$10,208	99.8%	100%
		23. Simulated to 80 years	$19,455	90%	96.7%
Overall	Based on the lifetable from the	24. Increased by 50% during post-	$30,878	74.8%	88.3%

© 2023 Liao CT et al. *JAMA Network Open*.

eTable 9. Assumptions in the Model and Relevant Scenario Analyses in the US Setting

Assumption	Original assumption	Scenario analyses	ICER value	Possibility of cost-effectiveness at the willingness-to-pay threshold of $50,000/QALY	Possibility of cost-effectiveness at the willingness-to-pay threshold of $100,000/QALY
mortality	U.S. statistics	trial period			
		25. Decreased by 50% during post-trial period	$17,097	98.2%	99.8%
Adverse event risk	Based on the STEP trial	26. Intensive treatment doubled the risk of adverse events	$31,765	77.6%	87.9%
		27. Risk of adverse events doubled in both groups	$25,259	88%	93%
Repeated CVD risk	Based on the STEP trial	28. Intensive treatment doubled the risk of recurrent CVD	$27,290	91.2%	90.6%
		29. Risk of recurrent CVD doubled in both groups	$21,710	92.2%	95.6%
**Supplementary Table S9.** Assumptions in the model and relevant scenario analyses in the U.S. setting					

© 2023 Liao CT et al. *JAMA Network Open*.

eTable 9. Assumptions in the Model and Relevant Scenario Analyses in the US Setting

Assumption	Original assumption	Scenario analyses	ICER value	Possibility of cost-effectiveness at the willingness-to-pay threshold of $50,000/QALY	Possibility of cost-effectiveness at the willingness-to-pay threshold of $100,000/QALY
Assumption	Original assumption	Scenario analyses	ICER value	Possibility of cost-effectiveness at the willingness-to-pay threshold of $50,000/QALY	Possibility of cost-effectiveness at the willingness-to-pay threshold of $100,000/QALY
Discount rate	Original value: 3%	30. Discount rate at 0	$18,372	97.1%	99.8%
		31. Discount rate at 5%	$30,044	73.9%	88.6%
Cycle length	Lifetime	32. Only trial period: four years	$144,225	38.8%	44.6%
		33. 10 years	$72,447	40.1%	58.1%
		34. 13 years (close to the U.S. life expectancy of 79.1 years)	$57,077	46.8%	71.9%
		35. 20 years	$36,297	66%	84.9%
		36. 30 years	$25,872	85.2%	93.2%

© 2023 Liao CT et al. *JAMA Network Open*.

eTable 10. Cost-effectiveness of Intensive vs Standard Blood Pressure Control Among Older Patients With Different Stratifications in the US

Variables	Subgroup	Primary-outcome yearly hazard ratio	ICER	PSA at different WTP thresholds	
			Cost per QALY gained	$50,000/QALY	$100,000/QALY
Age	60-69 years	0.75±0.26	$25,032	84.1%	93.6%
	70-80 years	0.74±0.37	$20,908	87.6%	95.6%
Sex	Men	0.71±0.30	$22,032	91.9%	94.8%
	Women	0.80±0.31	$28,982	79.8%	91.0%
Systolic blood pressure	≤138 mmHg	0.71±0.39	$21,136	87.1%	94.1%
	139–151 mmHg	0.95±0.36	$30,794	76.7%	90.3%
	≥152 mmHg	0.62±0.37	$22,024	90.9%	95.3%
Previous diabetes	Yes	0.78±0.42	$25,912	86.8%	94.1%
	No	0.74±0.25	$24,882	87.4%	94.5%
Blood-pressure measurement	App management	0.78±0.28	$25,912	87.0%	94.2%
	Usual management	0.72±0.33	$24,383	88.1%	94.6%

ICER, incremental cost-effectiveness ratio; QALY, quality-adjusted life-year; PSA, probabilistic sensitivity analyses

© 2023 Liao CT et al. *JAMA Network Open*.

eFigure 9. Probability of Cost-effectiveness of Intensive vs Standard Blood Pressure Control by Age, Sex, and Systolic Blood Pressure at Baseline in the US

A. Subgroups aged 60-69 and 70-80 years in the U.S.

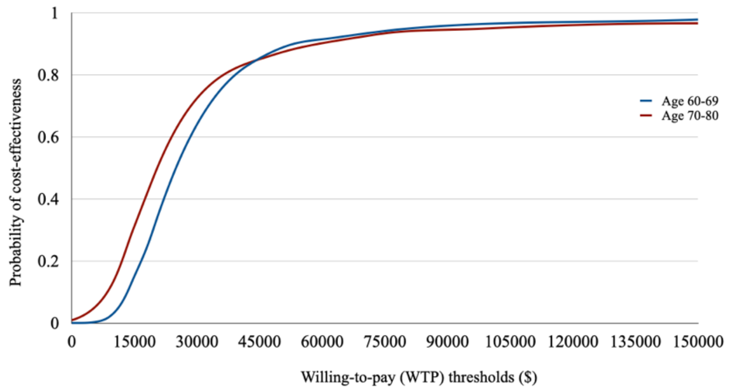

B. Male and female subgroups in the U.S.

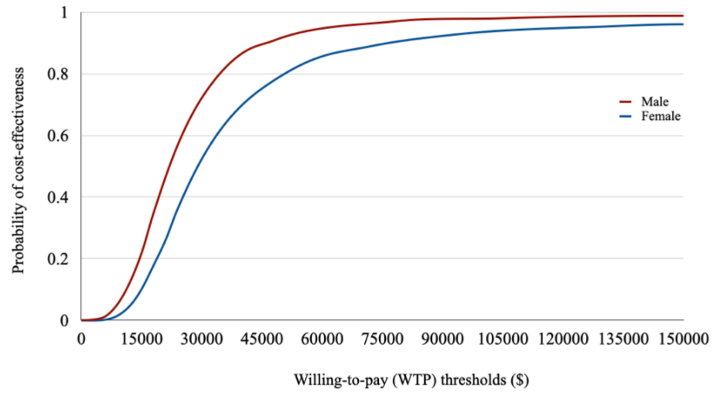

C. Different systolic pressure subgroups at baseline in the U.S.

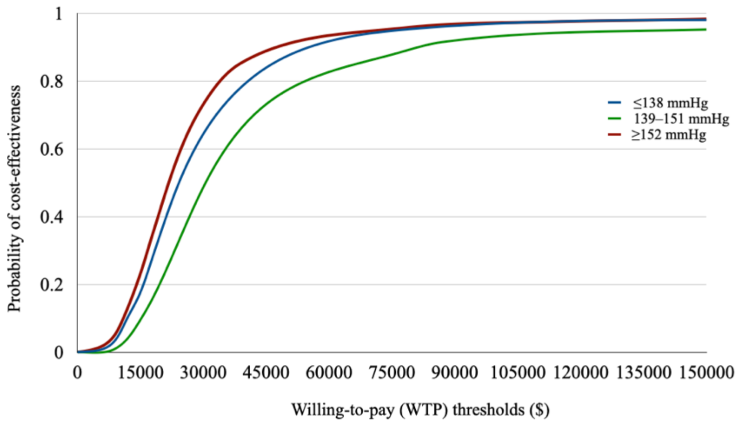

© 2023 Liao CT et al. *JAMA Network Open*.

eTable 11. Utility and Cost Parameters Simulated in the Model for the UK Setting

	Estimates	Range	Distribution	Reference
Utility				
Hypertension (age and sex dependent)	0.765	0.753-0.777	Uniform	Ara R et al.
Health event multiplier				
Acute coronary syndrome	0.76	0.52-0.88	Beta	Constanti M et al.
Stroke	0.628	0.42-0.84	Beta	Constanti M et al.
Post stroke	0.628	0.42-0.84	Beta	Constanti M et al.
Acute heart failure	0.683	0.46-0.87	Beta	Constanti M et al.
Atrial fibrillation	0.73	0.68-0.94	Beta	Sullivan PW et al.
Utility decrements				
Age per year	-0.003	-0.002 - -0.004	Beta	Liu X et al.
Chronic CHD	-0.12	-0.06 - -0.33	Beta	Li C et al.
Acute kidney injury	-0.323	-0.26 - -0.38	Beta	Constanti M et al. Applied for 4 weeks
Fracture	-0.343	-0.28 - -0.40	Beta	Constanti M et al. Applied for 12 weeks
Other adverse events	-0.1	-0.08 - -0.13	Beta	Bress AP et al. Applied for 2 weeks
Cardiovascular event costs (£)				
Acute coronary syndrome	3,420	1,710-5,129	Gamma	Constanti M et al., assumption
Stroke	18,081	95,91-28,773	Gamma	Patel A et al.
Coronary revascularization	5,661	2,830-8,491	Gamma	Leyva F et al., assumption
Heart failure	2,801	1,400-4,201	Gamma	Constanti M et al.,
Atrial fibrillation	731	366-1,097	Gamma	Burdett P et al. Estimated

© 2023 Liao CT et al. *JAMA Network Open*.

eTable 11. Utility and Cost Parameters Simulated in the Model for the UK Setting

	Estimates	Range	Distribution	Reference
Annual costs of cardiovascular states (£)				
Chronic coronary heart diseases	652	326-979	Gamma	Constanti M et al., assumption
Chronic Stroke	8,232	4,116-12,347	Gamma	Patel A et al.
Chronic heart failure	727	364-1,091	Gamma	Constanti M et al.,
Chronic atrial fibrillation	752	376-1,128	Gamma	Burdett P et al. Estimated
Adverse event costs (£)				
Hypotension	445	222-667	Gamma	Assume: one ER visit
Dizziness	445	222-667	Gamma	Assume: one ER visit
Syncope	1,711	856-2,267	Gamma	Adlan A.
Acute kidney injury	1,942	971-2,912	Gamma	Constanti M et al.,
Fracture	2,561	1,280-3,841	Gamma	Constanti M et al.,
Anti-hypertension related medical costs (£)				
Annual costs for intensive treatment	692	346-1,038	Gamma	Constanti M et al., assumption
Annual costs for standard treatment	534	267-801	Gamma	Constanti M et al., assumption
Other related medical costs (£)				
Background medical costs	3,684	1,842-5,525	Gamma	Office of National Statistics
Cardiovascular death	8,281	4,141-12,422	Gamma	Assumption
Non cardiovascular death	8,281	4,141-12,422	Gamma	Assumption

All costs were inflated to 2022. Assumption for costs of cardiovascular death was estimated by averaging the costs of acute coronary syndrome, stroke and heart failure. Assumption for costs of non-cardiovascular death was estimated as equal to cardiovascular death

© 2023 Liao CT et al. *JAMA Network Open*.

eFigure 10. Probability of Cost-effectiveness of Intensive vs Standard Blood Pressure Control in Different Adherence Scenarios in the UK

The curve presents the results after running 1000 simulations with random draws for all input parameters to capture joint uncertainty, and the probabilities of cost-effectiveness of intensive treatments changed with different willingness-to-pay (WTP) thresholds (costs in US$ per quality-adjusted life-year gained).

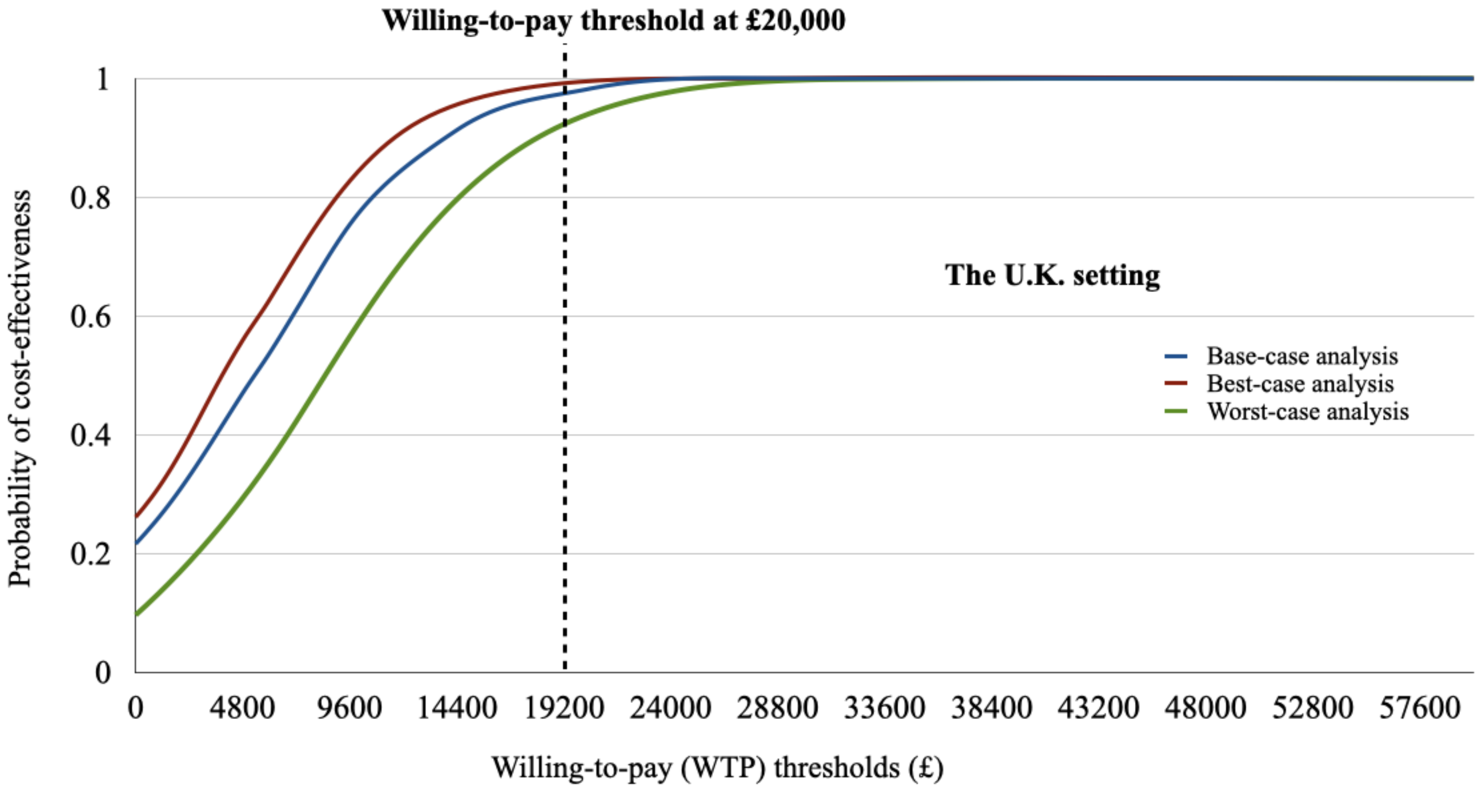

© 2023 Liao CT et al. *JAMA Network Open*.

eFigure 11. One-way Sensitivity Analysis Presented as a Tornado Diagram for the UK Setting.
ICER, incremental cost-effectiveness ratio. QALY, quality-adjusted life-year.

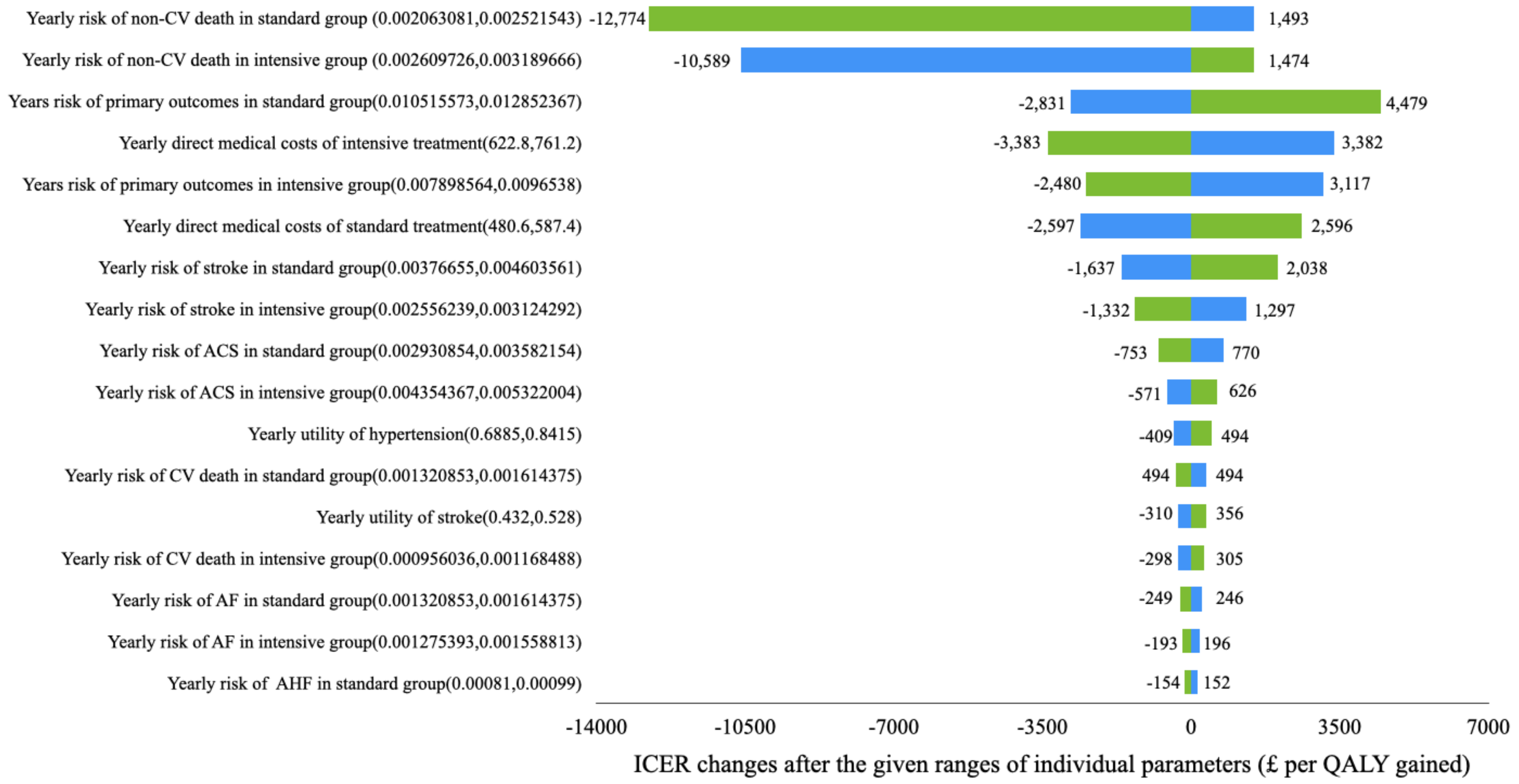

© 2023 Liao CT et al. *JAMA Network Open*.

Supplementary materials for scenario and subgroup analyses in the U.K.

eTable 12. Assumptions in the Model and Relevant Scenario Analyses in the UK Setting

Assumption	Original assumption	Scenario analyses	ICER value	Possibility of cost-effectiveness at the willingness-to-pay threshold of £20,000/QALY	Possibility of cost-effectiveness at the willingness-to-pay threshold of £30,000/QALY
Adherence	For base case, adherence in post-trial period for intensive versus standard control: 70% versus 75%	1. Adherence decreased to 60% for both groups	£5,107	98.9%	100%
		2. Adherence loss rates after trial period for intensive and standard treatment were assumed at 5% and 3% per year	£5,267	98.6%	100%
	For worst-case, adherence in post-trial period for intensive versus standard control: 0% versus 100%	3. Adherence decreased to 60% for standard control group	£8,353	95.6%	100%
	For best-case, adherence in post-trial period for intensive versus standard control: 100% versus 75%	4. Adherence decreased to 60% for standard control group	£3,350	99.7%	100%

© 2023 Liao CT et al. *JAMA Network Open*.

eTable 12. Assumptions in the Model and Relevant Scenario Analyses in the UK Setting

Assumption	Original assumption	Scenario analyses	ICER value	Possibility of cost-effectiveness at the willingness-to-pay threshold of £20,000/QALY	Possibility of cost-effectiveness at the willingness-to-pay threshold of £30,000/QALY
**Supplementary Table S12.** Assumptions in the model and relevant scenario analyses in the U.K. setting					
Assumption	Original assumption	Scenario analyses	ICER value	Possibility of cost-effectiveness at the willingness-to-pay threshold of £20,000/QALY	Possibility of cost-effectiveness at the willingness-to-pay threshold of £30,000/QALY
Costs	Intensive treatment annual costs (£692) and standard treatment annual costs (£541)	5. Doubled the intensive treatment annual costs (£1384)	£38,532	1.8%	48.1%
		6. 1.5 times the intensive treatment annual costs (£1038)	£21,617	40.7%	89.2%
		7. Intensive treatment annual costs 1.5 times standard treatment	£10,031	93.4%	100%
		8. Intensive treatment annual costs equal to standard treatment	-£3,022	Cost-saving	Cost-saving

© 2023 Liao CT et al. *JAMA Network Open*.

eTable 12. Assumptions in the Model and Relevant Scenario Analyses in the UK Setting

Assumption	Original assumption	Scenario analyses	ICER value	Possibility of cost-effectiveness at the willingness-to-pay threshold of £20,000/QALY	Possibility of cost-effectiveness at the willingness-to-pay threshold of £30,000/QALY
		9. Intensive treatment annual costs decreased to £595-596	£0	100% - cost-saving	100% - cost-saving
	CHD yearly costs (£635)	10. Double the costs (£1,270)	£4,341	99.4%	100%
		11. Half the costs (£318)	£4,883	99%	100%
	Acute stroke costs (£19,182)	12. Double the costs (£38,364)	£4,075	99.5%	100%
		13. Half the costs (£9,591)	£5,016	98.6%	100%

Supplementary Table S12. Assumptions in the model and relevant scenario analyses in the U.K. setting

Assumption	Original assumption	Scenario analyses	ICER value	Possibility of cost-effectiveness at the willingness-to-pay threshold of £20,000/QALY	Possibility of cost-effectiveness at the willingness-to-pay threshold of £30,000/QALY
Costs	Acute coronary syndrome costs (£3,420)	14. Double the costs. (£6,840)	£4,565	99.2%	100%
		15. Half the costs (£1,710)	£4,771	99.1%	100%
	Chronic stroke annual costs (£8,232)	16. Double the costs. (£16,464)	£2,548	99.2%	100%
		17. Half the costs. (£4,116)	£5,780	99.2%	100%

© 2023 Liao CT et al. *JAMA Network Open*.

eTable 12. Assumptions in the Model and Relevant Scenario Analyses in the UK Setting

Assumption	Original assumption	Scenario analyses	ICER value	Possibility of cost-effectiveness at the willingness-to-pay threshold of £20,000/QALY	Possibility of cost-effectiveness at the willingness-to-pay threshold of £30,000/QALY
Hazard ratio of primary outcomes during trial period	Hazard ratio: 0.74	18. Using the hazard ratio value (0.68), which was for patients aged 75 years and more in the SPRINT trial	£4,169	99.4%	100%
		19. Intensive control had the same hazard as standard control during trial period	£6,902	97.6%	100%
10-year CVD risk prediction model	Based on SCORE2/SCORE2-OP 10-year risk prediction	20. Increased by 50%	£3,109	100%	100%
		21. Decreased by 50%	£8,252	90%	100%
**Supplementary Table S12.** Assumptions in the model and relevant scenario analyses in the U.K. setting					
Assumption	Original assumption	Scenario analyses	ICER value	Possibility of cost-effectiveness at the willingness-	Possibility of cost-effectiveness at the willingness-

© 2023 Liao CT et al. *JAMA Network Open*.

eTable 12. Assumptions in the Model and Relevant Scenario Analyses in the UK Setting

Assumption	Original assumption	Scenario analyses	ICER value	Possibility of cost-effectiveness at the willingness-to-pay threshold of £20,000/QALY	Possibility of cost-effectiveness at the willingness-to-pay threshold of £30,000/QALY
				to-pay threshold of £20,000/QALY	to-pay threshold of £30,000/QALY
Overall mortality	Based on the lifetable from the U.K. statistics	22. Increased by 50% during post-trial period	£5,695	96.6%	100%
		23. Decreased by 50% during post-trial period	£3,672	100%	100%
Adverse event risk	Based on the STEP trial	24. Intensive treatment doubled the risk of adverse events	£5,487	98.1%	100%
		25. Risk of adverse events doubled in both groups	£4,697	99.4%	100%
Repeated CVD risk	Based on the STEP trial	26. Intensive treatment doubled the risk of recurrent CVD	£9,457	88.4%	99.3%

© 2023 Liao CT et al. *JAMA Network Open*.

eTable 12. Assumptions in the Model and Relevant Scenario Analyses in the UK Setting

Assumption	Original assumption	Scenario analyses	ICER value	Possibility of cost-effectiveness at the willingness-to-pay threshold of £20,000/QALY	Possibility of cost-effectiveness at the willingness-to-pay threshold of £30,000/QALY
		27. Risk of recurrent CVD doubled in both groups	£6,512	95.7%	100%
**Supplementary Table S12.** Assumptions in the model and relevant scenario analyses in the U.K. setting					
Assumption	Original assumption	Scenario analyses	ICER value	Possibility of cost-effectiveness at the willingness-to-pay threshold of £20,000/QALY	Possibility of cost-effectiveness at the willingness-to-pay threshold of £30,000/QALY
Discount rate	Original value: 3%	28. Discount rate at 0	£3,318	99.8%	100%
		29. Discount rate at 5%	£5,463	97.9%	100%
Cycle length	Lifetime	30. Only trial period: four years	£34,207	41.4%	48.7%
		31. 10 years	£13,987	60.5%	87.2%
		32. 15 years (close to the U.K. life expectancy of 81.65 years)	£8,843	82.3%	99.9%
		33. 20 years	£6,388	95.8%	100%

© 2023 Liao CT et al. *JAMA Network Open*.

eTable 12. Assumptions in the Model and Relevant Scenario Analyses in the UK Setting

Assumption	Original assumption	Scenario analyses	ICER value	Possibility of cost-effectiveness at the willingness-to-pay threshold of £20,000/QALY	Possibility of cost-effectiveness at the willingness-to-pay threshold of £30,000/QALY
		34. 30 years	£4,801	98.8%	100%

eTable 13. Cost-effectiveness of Intensive vs Standard Blood Pressure Control Among Older Patients With Different Stratifications in the UK Setting

Variables	Subgroup	Primary-outcome yearly hazard ratio	ICER	PSA at different WTP thresholds	
			Cost per QALY gained	£20,000/QALY	£30,000/QALY
Age	60-69 years	0.75±0.26	£4,671	99.2%	100%
	70-80 years	0.74±0.37	£5,035	96.8%	100%
Sex	Men	0.71±0.30	£4,389	99.4%	100%
	Women	0.80±0.31	£5,091	98.6%	100%

© 2023 Liao CT et al. *JAMA Network Open*.

Systolic blood pressure	≤138 mmHg	0.71±0.39	£4,390	99.3%	100%
	139–151 mmHg	0.95±0.36	£6,412	97.1%	100%
	≥152 mmHg	0.62±0.37	£3,747	99.7%	100%
Previous diabetes	Yes	0.78±0.42	£4,930	99.1%	100%
	No	0.74±0.25	£4,617	99.3%	100%
Blood-pressure measurement	App management	0.78±0.28	£4,930	98.8%	100%
	Usual management	0.72±0.33	£4,465	99.4%	100%

ICER, incremental cost-effectiveness ratio; QALY, quality-adjusted life-year; PSA, probabilistic sensitivity analyses

© 2023 Liao CT et al. *JAMA Network Open*.

eFigure 12. Probability of Cost-effectiveness of Intensive vs Standard Blood Pressure Control by Age, Sex, and Systolic Blood Pressure at Baseline in the UK

A. Subgroups aged 60-69 and 70-80 years in the U.K.

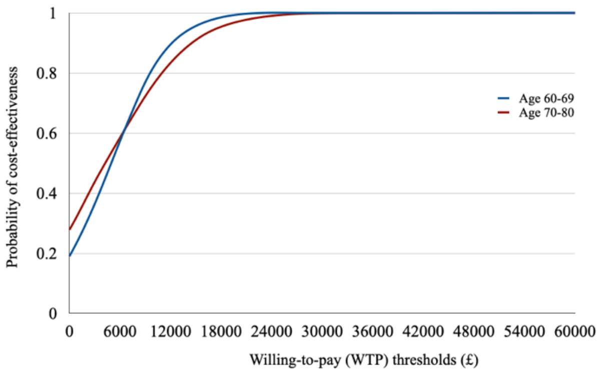

B. Male and female subgroups in the U.K.

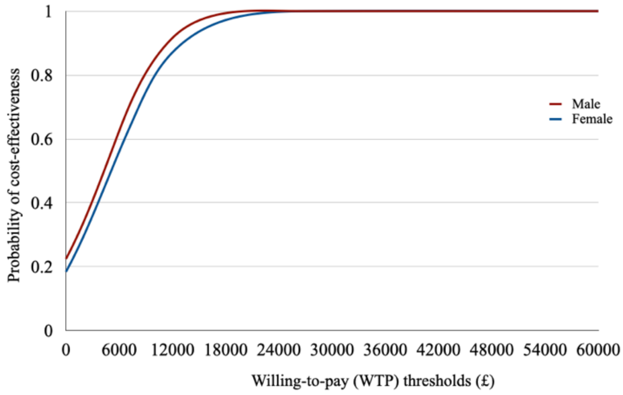

C. Different systolic pressure subgroups at baseline in the U.K.

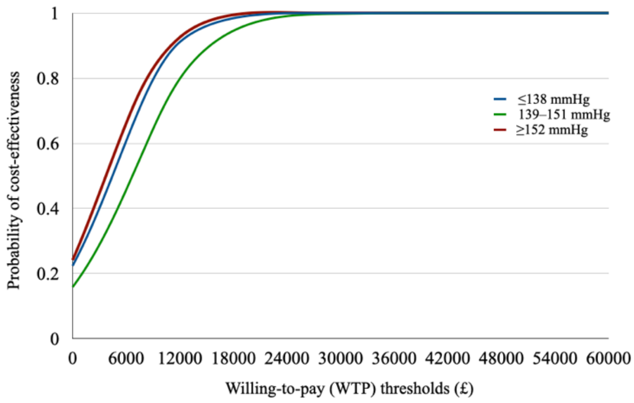

© 2023 Liao CT et al. *JAMA Network Open*.

eReferences

1. Iskedjian M, Einarson TR, MacKeigan LD, et al. Relationship between daily dose frequency and adherence to antihypertensive pharmacotherapy: evidence from a meta-analysis. Clin Ther. 2002 Feb;24(2):302-16.
2. Liu X, Bai G, Li H, Li S. Applying SF-6D to measure health state utilities among the middle and old aged patients with hypertension in China. Health Qual Life Outcomes. 2020 Dec 11;18(1):385.
3. Li C, Chen K, Cornelius V, Tomeny E, Wang Y, Yang X, Yuan X, Qin R, Yu D, Wu Z, Wang D, Chen T. Applicability and cost-effectiveness of the Systolic Blood Pressure Intervention Trial (SPRINT) in the Chinese population: A cost-effectiveness modeling study. PLoS Med. 2021 Mar 4;18(3):e1003515.
4. Bress AP, Bellows BK, King JB, Hess R, Beddhu S, Zhang Z, Berlowitz DR, Conroy MB, Fine L, Oparil S, Morisky DE, Kazis LE, Ruiz-Negrón N, Powell J, Tamariz L, Whittle J, Wright JT Jr, Supiano MA, Cheung AK, Weintraub WS, Moran AE; SPRINT Research Group. Cost-Effectiveness of Intensive versus Standard Blood-Pressure Control. N Engl J Med. 2017 Aug 24;377(8):745-755.
5. Harrington AR, Armstrong EP, Nolan PE Jr, Malone DC. Cost-effectiveness of apixaban, dabigatran, rivaroxaban, and warfarin for stroke prevention in atrial fibrillation. Stroke. 2013 Jun;44(6):1676-81.
6. Constanti M, Floyd CN, Glover M, Boffa R, Wierzbicki AS, McManus RJ. Cost-Effectiveness of Initiating Pharmacological Treatment in Stage One Hypertension Based on 10-Year Cardiovascular Disease Risk: A Markov Modeling Study. Hypertension. 2021 Feb;77(2):682-691.
7. Hu S, Zhan L, Liu B, Gao Y, Li Y, Tong R, Wu L, Yu B, Gao S. Economic Burden of Individual Suffering from Atrial Fibrillation-Related Stroke in China. Value Health Reg Issues. 2013 May;2(1):135-140.
8. Wei H, Cui C, Cui X, Liu Y, Li D. Cost-effectiveness analysis of dabigatran, rivaroxaban and warfarin in the prevention of stroke in patients with atrial fibrillation in China. BMC Health Serv Res. 2021 Jan 28;21(1):96.
9. Liu P, Ma S, Du G, Sun S, Zhang X, Tang P, Hou C, Liu Y, Zhao J, Zhang X, Chen L, Gu C, Zhang L, Chong L, Yang X, Li R. Changing Paradigm for Vertigo/Dizziness Patients: a Retrospective Before-After Study from Tertiary Hospitals in Northwestern China. J Gen Intern Med. 2021 Oct;36(10):3064-3070.
10. Li YW, Chen L, Du JB, Yang YY, Jin HF. Cost-effectiveness of diagnostic approaches to vasovagal syncope. Chin Med J (Engl). 2010 Oct;123(19):2635-9.
11. Fang Y, Ding X, Zhong Y, Zou J, Teng J, Tang Y, Lin J, Lin P. Acute kidney injury in a Chinese hospitalized population. Blood Purif. 2010;30(2):120-6.

© 2023 Liao CT et al. *JAMA Network Open*.

12. Yang Y, Du F, Ye W, Chen Y, Li J, Zhang J, Nicely H, Burge R. Inpatient cost of treating osteoporotic fractures in mainland China: a descriptive analysis. Clinicoecon Outcomes Res. 2015 Apr 13;7:205-12.
13. Yang L, Wu M, Cui B, Xu J. Economic burden of cardiovascular diseases in China. Expert Rev Pharmacoecon Outcomes Res. 2008 Aug;8(4):349-56.
14. Fryback DG, Dunham NC, Palta M, Hanmer J, Buechner J, Cherepanov D, Herrington SA, Hays RD, Kaplan RM, Ganiats TG, Feeny D, Kind P. US norms for six generic health-related quality-of-life indexes from the National Health Measurement study. Med Care. 2007 Dec;45(12):1162-70.
15. Stein JD, Brown GC, Brown MM, Sharma S, Hollands H, Stein HD. The quality of life of patients with hypertension. J Clin Hypertens (Greenwich). 2002 May-Jun;4(3):181-8.
16. Liu X, Bai G, Li H, Li S. Applying SF-6D to measure health state utilities among the middle and old aged patients with hypertension in China. Health Qual Life Outcomes. 2020 Dec 11;18(1):385.
17. Nagle PC, Smith AW. Review of recent US cost estimates of revascularization. Am J Manag Care. 2004 Oct;10(11 Suppl):S370-6.
18. Saber Tehrani AS, Coughlan D, Hsieh YH, Mantokoudis G, Korley FK, Kerber KA, Frick KD, Newman-Toker DE. Rising annual costs of dizziness presentations to U.S. emergency departments. Acad Emerg Med. 2013 Jul;20(7):689-96.
19. Bonafede M, Espindle D, Bower AG. The direct and indirect costs of long bone fractures in a working age US population. J Med Econ. 2013;16(1):169-78.
20. Peter G. PETERSON FOUNDATION. WHY ARE AMERICANS PAYING MORE FOR HEALTHCARE? Access on 20 March 2022. https://www.pgpf.org/blog/2022/02/why-are-americans-paying-more-for-healthcare
21. French EB, McCauley J, Aragon M, Bakx P, Chalkley M, Chen SH, Christensen BJ, Chuang H, Côté-Sergent A, De Nardi M, Fan E, Échevin D, Geoffard PY, Gastaldi-Ménager C, Gørtz M, Ibuka Y, Jones JB, Kallestrup-Lamb M, Karlsson M, Klein TJ, de Lagasnerie G, Michaud PC, O'Donnell O, Rice N, Skinner JS, van Doorslaer E, Ziebarth NR, Kelly E. End-Of-Life Medical Spending In Last Twelve Months Of Life Is Lower Than Previously Reported. Health Aff (Millwood). 2017 Jul 1;36(7):1211-1217.
22. Ara R, Brazier JE. Using health state utility values from the general population to approximate baselines in decision analytic models when condition-specific data are not available. Value Health. 2011 Jun;14(4):539-45.
23. Sullivan PW, Ghushchyan V. Preference-Based EQ-5D index scores for chronic conditions in the United States. Med Decis Making. 2006 Jul-Aug;26(4):410-20.
© 2023 Liao CT et al. *JAMA Network Open*.

24. Patel A, Berdunov V, Quayyum Z, King D, Knapp M, Wittenberg R. Estimated societal costs of stroke in the UK based on a discrete event simulation. Age Ageing. 2020 Feb 27;49(2):270-276.
25. Leyva F, Qiu T, Evison F, Christoforou C, McNulty D, Ludman P, Ray D. Clinical outcomes and costs of cardiac revascularisation in England and New York state. Open Heart. 2018 Jan 3;5(1):e000704.
26. Burdett P, Lip GYH. Atrial fibrillation in the UK: predicting costs of an emerging epidemic recognizing and forecasting the cost drivers of atrial fibrillation-related costs. Eur Heart J Qual Care Clin Outcomes. 2022 Mar 2;8(2):187-194.
27. Adlan A. Structured Approach in Syncope. Access on 21 March 2022. https://www.britishcardiovascularsociety.org/resources/editorials/articles/pci-for-lv-dysfunction
28. Office of National Statistics. Healthcare expenditure, UK Health Accounts: 2019. Access on 23 March 2022. https://www.ons.gov.uk/peoplepopulationandcommunity/healthandsocialcare/healthcaresystem/bulletins/ukhealthaccounts/2019
29. Liu X, Bai G, Li H, Li S. Applying SF-6D to measure health state utilities among the middle and old aged patients with hypertension in China. Health Qual Life Outcomes. 2020 Dec 11;18(1):385.
30. Harrington AR, Armstrong EP, Nolan PE Jr, Malone DC. Cost-effectiveness of apixaban, dabigatran, rivaroxaban, and warfarin for stroke prevention in atrial fibrillation. Stroke. 2013 Jun;44(6):1676-81.
31. Constanti M, Floyd CN, Glover M, Boffa R, Wierzbicki AS, McManus RJ. Cost-Effectiveness of Initiating Pharmacological Treatment in Stage One Hypertension Based on 10-Year Cardiovascular Disease Risk: A Markov Modeling Study. Hypertension. 2021 Feb;77(2):682-691.
32. Hu S, Zhan L, Liu B, Gao Y, Li Y, Tong R, Wu L, Yu B, Gao S. Economic Burden of Individual Suffering from Atrial Fibrillation-Related Stroke in China. Value Health Reg Issues. 2013 May;2(1):135-140.
33. Wei H, Cui C, Cui X, Liu Y, Li D. Cost-effectiveness analysis of dabigatran, rivaroxaban and warfarin in the prevention of stroke in patients with atrial fibrillation in China. BMC Health Serv Res. 2021 Jan 28;21(1):96.

© 2023 Liao CT et al. *JAMA Network Open*.
