# Supplementary material for: Cost-effectiveness of Intensive vs Standard Blood Pressure Control Among Older Patients With Hypertension
Source: JAMA Netw Open. 2023 Feb 27;6(2):e230708. doi: 10.1001/jamanetworkopen.2023.0708 (PMC9972197; doi:10.1001/jamanetworkopen.2023.0708)
Supplement: Supplement 2. — Data Sharing Statement [file jamanetwopen-e230708-s002.pdf]

Data Sharing Statement

Liao. Cost-effectiveness of Intensive vs Standard Blood Pressure Control Among Older Patients With Hypertension. *JAMA Netw Open*. Published February 27, 2023. doi:10.1001/jamanetworkopen.2023.0708

Data
Data available: Yes
Data types: Deidentified participant data
How to access data: The step trial data is publicly accessible.
When available: With publication

Supporting Documents
Document types: None

Additional Information
Who can access the data: Researchers whose proposed use of the data has been approved
Types of analyses: For any purpose or for a specified purpose
Mechanisms of data availability: With investigator support.
